# Cell type-specific genome scans of DNA methylation divergence indicate an important role for transposable elements

**DOI:** 10.1186/s13059-020-02068-2

**Published:** 2020-07-13

**Authors:** Önder Kartal, Marc W. Schmid, Ueli Grossniklaus

**Affiliations:** 1 Department of Plant and Microbial Biology & Zurich-Basel Plant Science Center, University of Zurich, Zollikerstrasse 107, Zurich, 8008 Switzerland; 2 Creoptix AG, Zugerstrasse 76, Wädenswil, 8820 Switzerland; 3 MWSchmid GmbH, Möhrlistrasse 25, Zurich, 8006 Switzerland

**Keywords:** Epigenomics, Population biology, Information theory, Jensen-Shannon divergence

## Abstract

In population genomics, genetic diversity measures play an important role in genome scans for divergent sites. In population epigenomics, comparable tools are rare although the epigenome can vary at several levels of organization. We propose a model-free, information-theoretic approach, the Jensen-Shannon divergence (JSD), as a flexible diversity index for epigenomic diversity. Here, we demonstrate how JSD uncovers the relationship between genomic features and cell type-specific methylome diversity in *Arabidopsis thaliana*. However, JSD is applicable to any epigenetic mark and any collection of individuals, tissues, or cells, for example to assess the heterogeneity in healthy organs and tumors.

## Background

The ongoing development of sequencing-based functional genomics has a tremendous impact on the study of gene regulation. Nowadays, we can get an almost comprehensive, genome-wide readout of gene expression and chromatin states. This technological progress not only produces genomic data sets for more and more organisms but enables us to profile gene regulation at the level of organs, tissues, and even individual cells. However, new technologies beget new problems. We are confronted with multidimensional data sets and a sophisticated sampling situation that involves population structure, cell heterogeneity, temporal change, and technical bias, raising new questions about how regulatory factors vary within and across these different levels. The measurement of diversity and its apportionment is prominent in population biology to assess species or genetic diversity. In population genomics, for example, genome-wide scans with measures of genetic differentiation are used to detect conserved or polymorphic sites [1]. In contrast, population-level measures to perform genome-wide scans for epigenetically divergent sites are still missing but the data that are now available provide a solid foundation for developing and testing such measures.

DNA methylation has been studied extensively in humans and several model organisms at genome scale. Whole-genome sequencing of bisulfite-converted DNA (BS-seq) [2–4] provides accurate, genome-wide maps of the chemically modified cytosine base 5-methylcytosine (5mC) at single-base resolution, so-called methylomes. Although 5mC is not ubiquitous, it is widespread in higher eukaryotes [5]. In vertebrates, it is mainly found at CpG dinucleotides (CG context), whereas plants harbor 5mC also in the CHG and CHH context (H being A, C, or T).

DNA methylation can interfere with gene expression and contributes to the silencing of repetitive elements [6]. Moreover, the DNA methylation landscape must be actively controlled throughout the life cycle by an enzymatic machinery. In mammals, extensive reprogramming takes place during primordial germ cell development and early embryogenesis [7] and the methylome also changes during tumorigenesis [8]. In plants, reprogramming is less extensive and the details are yet unclear but some epigenetic marks get reprogrammed during reproduction [9–11]. A drastic perturbation of the methylation pathways is lethal to mammalian embryos [12] and can lead to sterility and developmental aberrations after inbreeding in plants [13]. These severe effects illustrate an essential role for DNA methylation, not only for the activity of specific genes but for the integrity of the genome as a whole.

Due to its correlation with fitness-relevant traits [14, 15] and its susceptibility to stress [16], DNA methylation has attracted considerable interest in evolutionary biology as a mediator of soft inheritance [17]. For evolutionary studies, *Arabidopsis thaliana* is an excellent model for organisms with a full-featured methylation machinery. It has a small genome, a short life cycle, and large populations that harbor methylation polymorphisms of natural [15, 18–20] as well as artificial origin [21, 22] are available.

**Fig. 1:**
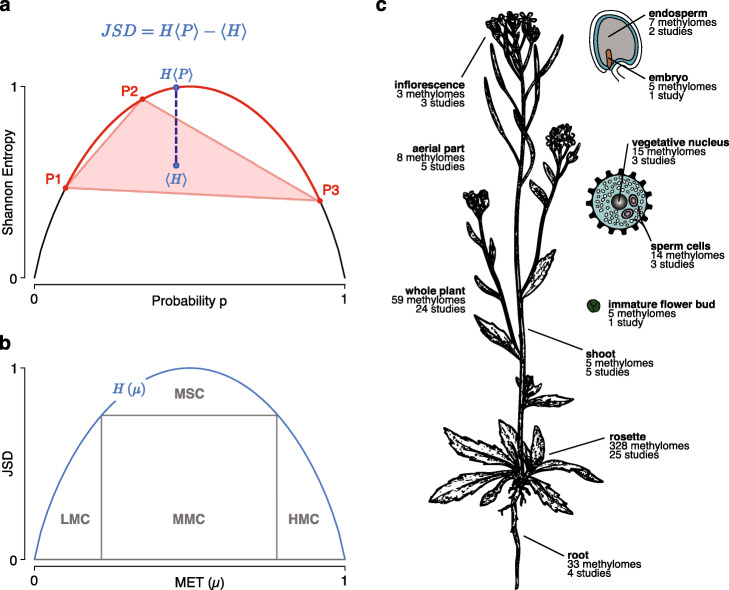
Application of Jensen-Shannon divergence to DNA methylation. **a** Geometric explanation of Jensen-Shannon divergence (JSD) in terms of Shannon entropy. The graph shows the entropy of a binary distribution (with probabilities *p* and *q*=1−*p*) in terms of *p*. For the purpose of illustration, the red dots designate the coordinates of three distributions, *P*_1_,*P*_2_,*P*_3_, that define a red curve segment and a corresponding polygon (red triangle). The curve segment constrains the entropy of the mixture distribution, *H*〈*P*〉, while the polygon constrains the corresponding average entropy, 〈*H*〉. The exact location of both JSD terms depends on the weights. For equally weighted distributions (here *π*_*i*_=1/3 for all *i*), their locations are given by the blue dots. The corresponding distance (length of the dashed line) equals JSD. **b** Phase plane in terms of weighted average methylation MET (*μ*) and diversity index JSD. The methylation state of a cytosine in the population is represented by a point at or below the graph. Four regions of interest are highlighted: three regions with JSD below ≈0.7, LMC (low-methylated cytosines), MMC (medium-methylated cytosines), and HMC (high-methylated cytosines); a region with high JSD for MSCs (metastable cytosines). **c** Overview of methylome data sources. Four hundred eighty-two *Arabidopsis thaliana* methylomes from 75 different studies have been analyzed. All methylomes derive from wild-type plants of the Columbia 0 (Col-0) accession. The image is modified based on [26]

To enable genome scans of DNA methylation divergence at single-base resolution, we use a non-parametric approach based on Jensen-Shannon divergence (JSD), a divergence measure in information theory with unique properties [23, 24]. JSD measures the loss of information (or, equivalently, the increase in uncertainty) if a set of distinct, information-carrying units is pooled. To define JSD formally, the concepts of probability distribution and Shannon entropy [25] are necessary (see the “Methods” section for the details) but Fig. 1a illustrates the calculation of JSD geometrically. In this example, the three probability distribution functions (PDFs) are a sample from the population whose divergence is estimated by measuring the length of the dashed blue line. In the case of methylome data, the PDF of each individual is derived from the count data in a methylation table. A methylation table assigns two numbers to each cytosine site in the reference genome, the count of methylated and unmethylated reads. Therefore, in a population sample with *s* methylomes, each site is associated with a contingency table of 2×*s* entries. JSD is used to map this site-specific table to a site-specific number that reflects whether the methylation state at the given site is conserved (dip in JSD) or diversified (peak in JSD). More details and an example are given in the “Methods” section.

We have analyzed DNA methylation divergence in different parts of *Arabidopsis thaliana* as summarized in Fig. 1c. We distinguish the methylomes according to the source of the corresponding DNA, that is according to which cell type, tissue, or organ the DNA was extracted from. Our analysis emphasizes the role of sequence context, chromatin accessibility, and genomic location, particularly the proximity to transposable elements (TEs), in shaping DNA methylation divergence.

## Results

### Genomic spectrum of methylation divergence

This section summarizes the genome-wide features of DNA methylation divergence depending on genomic source and sequence (C) context. The source is expected to affect methylation divergence because the activity of genes differs between tissues and cell types. The C context is expected to affect divergence because different mechanisms are responsible for maintaining methylation in the CG, CHG, and CHH context [27].

#### Divergence is higher in the CG than the non-CG context

The “phase plane” spanned by MET and JSD for each C-site (see Fig. 1b and the “Methods” section) provides a bird’s-eye view of methylome divergence in a population. Also, as indicated in Fig. 1b, we have found it useful to distinguish the sites into different cytosine types (C types), based on their position in the phase plane: first of all, we distinguish between low-methylated Cs (MET <0.2, abbreviated as LMCs) and high-methylated Cs (MET >0.8, abbreviated as HMCs). These boundaries are not set in stone and just conform to our observations that Cs in unmethylated and methylated regions, respectively, often fall into these intervals. LMCs and HMCs differ little among each other in their MET values, respectively, but can differ substantially in their JSD values. Due to the entropy upper bound, LMCs and HMCs can have a JSD between 0 and ≈0.72 bit, respectively. The remaining sites, falling into the region 0.2≤MET≤0.8, are not as easy to characterize as the previous ones. This interval contains a subregion with sites that have a range of JSD similar to LMCs and HMCs. We denote these as medium-methylated Cs (MMCs) in order to distinguish them from sites with exceptionally high JSD above 0.7 bit, which we classify as metastable Cs (MSCs). MSCs are special because at these sites, the population tends to segregate into two subpopulations, methylated and unmethylated. In the following, we have used the classification into C types to analyze their global (i.e., whole-genome) as well as local enrichment using non-overlapping, genomic intervals along the genome.

**Fig. 2:**
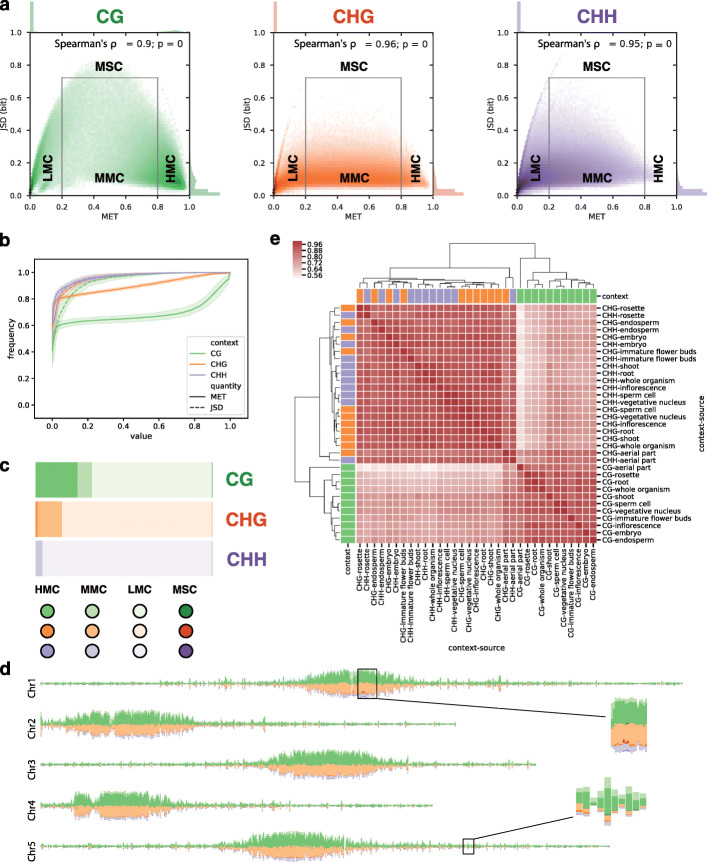
Genomic spectrum of methylation divergence. **a** Methylome phase plane for rosette leaves and all three sequence contexts. The C context colormap is used throughout the paper. In the margins, the distribution of MET and JSD, respectively, are shown. SciPy (scipy.stats.spearmanr) was used to compute Spearman’s *ρ* and the corresponding *p* value. **b** Empirical cumulative distribution function for MET and JSD in all three C contexts (same color code as phase plane). The line and error band display the mean and standard deviation of the data of all sources. **c** Proportions of C types for rosette leaves based on phase plane partitioning. The color hue indicates the different C types for each C context. **d** Rosette chromosome tracks for the proportions of C types following the color code in subfigure c. Bars for all C types except LMCs are stacked at non-overlapping, 50-kb intervals over the nuclear chromosomes (1 to 5 from top to bottom). Since LMCs are not explicitly shown, a smaller stack implicates a high proportion of LMCs. **e** Hierarchical clustering of genomic JSD signal for all source-context combinations using Spearman’s *ρ* over non-overlapping, 50-kb intervals

Figure 2a shows the rosette leaf phase plane for each context. It illustrates what we find in all analyzed sources, namely that the methylome is stable over a wide range of MET values. Both the histograms in the margins of the phase plane plots in Fig. 2a and the empirical cumulative distribution functions in Fig. 2b show that more than 90% of C-sites have a JSD below 0.2 bit regardless of C context. The genome-wide proportions of C types in all three C contexts are depicted in Fig. 2c, again for rosette leaves only. Table 1, however, gives a detailed overview of C-type proportions in all methylome sources: MSCs make up only a very small fraction of C-sites and the majority are LMCs regardless of C context, as expected for the largely unmethylated genome of *Arabidopsis thaliana*. But a closer look at the remaining C types reveals that HMCs tend to be enriched in the CG context while MMCs tend to be enriched in the CHG context.

**Table 1:** Genome-wide proportions of C types per source and context

		Proportion (%)		
	Context	CG	CHG	CHH
Source	C type			
Aerial part	HMC	26.25	2.87	0.25
	LMC	65.41	81.99	92.41
	MMC	7.78	14.59	6.45
	MSC	0.54	0.53	0.87
Embryo	HMC	23.48	4.61	0.54
	LMC	72.07	86.67	93.31
	MMC	4.15	8.20	5.75
	MSC	0.28	0.49	0.38
Endosperm	HMC	11.69	2.56	0.11
	LMC	73.21	87.94	95.90
	MMC	14.00	8.80	3.65
	MSC	1.08	0.67	0.32
Flower buds	HMC	30.24	7.00	0.32
	LMC	63.68	82.12	94.27
	MMC	5.67	10.31	5.04
	MSC	0.40	0.55	0.35
Inflorescence	HMC	25.42	3.49	0.02
	LMC	69.91	85.30	97.43
	MMC	4.65	11.18	2.52
	MSC	0.00	0.01	0.00
Root	HMC	21.53	1.00	0.01
	LMC	69.12	84.85	96.73
	MMC	9.10	14.12	3.24
	MSC	0.23	0.01	0.00
Rosette	HMC	23.84	1.22	0.02
	LMC	67.75	84.83	96.01
	MMC	8.21	13.91	3.95
	MSC	0.18	0.02	0.00
Shoot	HMC	21.08	1.63	0.13
	LMC	68.29	83.56	94.82
	MMC	10.56	14.76	4.97
	MSC	0.05	0.03	0.06
Sperm cell	HMC	29.99	2.13	0.00
	LMC	67.87	82.21	99.35
	MMC	2.12	15.62	0.64
	MSC	0.00	0.02	0.00
Vegetative nucleus	HMC	23.18	7.23	0.32
	LMC	70.60	83.44	94.88
	MMC	6.17	9.28	4.77
	MSC	0.03	0.02	0.02
Whole organism	HMC	23.51	1.44	0.01
	LMC	68.22	83.67	95.32
	MMC	8.21	14.86	4.64
	MSC	0.04	0.01	0.01

**Fig. 3:**
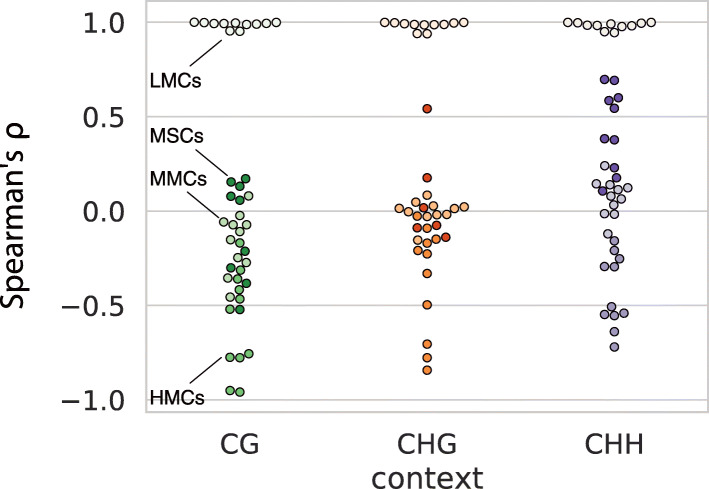
Correlation between MET and JSD. Spearman’s *ρ* (*y*-axis) was measured in each subregion of the phase plane, defining a C type (encoded by hue), and for each C context (encoded by color and *x*-axis)

The dominance of LMCs is responsible for the overall positive correlation between MET and JSD, as measured by Spearman’s rank-order correlation coefficient *ρ* (hereinafter abbreviated by Spearman’s *ρ*), see Fig. 2a. However, this obscures the more complex picture if we look at different subregions of the phase plane. Figure 3 depicts Spearman‘s *ρ* between MET and JSD for different C types for all combinations of source and context. The correlation is close to 1 for LMCs, regardless of source and context, whereas the positive correlation is not observed in other C types: for HMCs, MET and JSD are rather negatively correlated; for MMCs, MET and JSD are virtually uncorrelated; and for MSCs, the correlation is around zero (CG context) or only weakly positive (non-CG context).

As shown in Fig. 2b, the cumulative distribution functions of MET and JSD, respectively, do not differ by C context up to the median value of the distribution (specified at frequency =0.5). However, there is a tendency for higher JSD in the CG context indicated by the long tails of the distributions at higher percentiles, respectively. The Mann-Whitney *U* test has confirmed that there is strong evidence for higher JSD in the CG context (Additional file 1: Table S2). The propensity for higher MET in the CG context is also reflected by the different proportions of C types: around 24% of the CG sites are HMCs, as opposed to only around 1% of the CHG and only 0.02% of the CHH sites (see Fig. 2c and Table 1 for a comprehensive summary). In fact, the joint distribution in the rosette phase plane for CGs is bimodal (see histograms in the margins of Fig. 2a, left) with one peak at LMCs and another one with slightly higher JSD formed by HMCs. The rosette phase plane demonstrates another striking difference between the CG and CHG (and, to some extent, CHH) context around MET =0.5: non-CG sites mainly occupy regions of lower JSD meaning that these sites show consistently intermediate levels of methylation in the majority of individuals. The reason for these intermediate MET values is unknown and needs to be further analyzed. These sites could be methylated allele-specifically, but with our data, we cannot confirm this because the tissue-specific methylomes are mixtures of different cells (and cell types). Thus, intermediate methylation signals could be the result of pooling many cells.

Note that the 328 rosette methylomes considered here originate from leaves that have been harvested at different developmental stages (9), from plants that have been grown in different labs (13), under different stresses (7), and photoperiods (3). The stability of the methylome despite this heterogeneity suggests that environmental conditions have a minor effect on methylation at large and that the genotype and cell type-specific regulation may play the main role in shaping the methylation landscape [28].

#### Heterochromatin is enriched in CG-HMCs and CHG-MMCs

The chromosome tracks for the proportions of C types in rosettes (Fig. 2d, see Additional file 1: Fig. S1 for all sources) show increasing proportions of methylated sites in regions that are rich in repetitive elements and usually heterochromatic: the pericentromeric regions and, for example, the knob in the left arm of chromosome 4. The chromosome arms dominated by protein-coding genes show enrichment for LMCs. This pattern of methylation is in accordance with well-established findings for *Arabidopsis thaliana* [3, 4]. However, we can observe that pericentromeric chromatin is dominated by CG-HMCs and CHG-MMCs and that differences between the methylome sources are mainly due to shifts between HMCs and MMCs. In the CG context, HMCs are dominant in all sources but the endosperm. In the CHG context, HMCs are virtually absent in roots and rosettes; slightly increased in embryo, endosperm, and sperm cells; and on a par with MMCs in the vegetative nucleus and flower buds. In the CHH context, HMCs are virtually absent in all sources. MMCs form the smallest fraction of (partially) methylated sites in all sources and are virtually absent in sperm cells. The lack of MMCs in the haploid sperm cells concurs with our interpretation of MMCs given above since heterozygosity is not possible in haploid cells. MSCs also form only a tiny fraction of Cs in the heterochromatic regions with no substantial difference between the sources, although MSCs appear to be slightly increased in endosperm. In contrast to the regions dominated by heterochromatin, there are no regions in the chromosome arms that have a particularly high proportion of MSCs. In conclusion, the enrichment of metastable sites in heterochromatin suggests that this is due to the neutrality of single cytosine polymorphisms in transcriptionally silenced regions.

#### The source determines JSD in the non-CG context

Although genome-wide there is a positive correlation between MET and JSD, we have seen that these quantities can become uncoupled in certain regions of the phase plane and, thus, reflect different properties of the methylome. Hence, we assumed that comparing all combinations of source and context by MET or JSD may lead to different results. By correlating the mean at non-overlapping, 10-kb genomic intervals, we have performed a hierarchical clustering for JSD (Fig. 2e) and for MET (Additional file 1: Fig. S2). First, for both genomic signals, there is a clear separation into CG and non-CG context. In the non-CG context, however, the MET signals cluster differently than the JSD signals: while context still separates the signals before the source in the case of MET (with the exception of embryo and endosperm, which cluster by source first and then by context, respectively), JSD separates by source first and then by context. Here, the exceptions are root, vegetative nucleus, and sperm cell: all three cluster together in the CHG context, but in the CHH context, only vegetative nucleus and sperm cluster together, while roots are more similar to rosettes. We think that the general pattern observed here reflects the interplay between maintenance and de novo methylation.

There is a robust mechanism in place to maintain CG methylation across cell divisions [29], such that the strong similarity across tissues and cell types is plausible. On the other hand, it has been observed that changes in non-CG methylation accompany cell differentiation, which suggests the effect of de novo methylation [30]. In contrast to the strong separation into CG and non-CG context, the merely weak separation into CHG and CHH context along the MET coordinate is overridden along the JSD coordinate through a clear separation into different sources. This may reflect the importance of cell or tissue differentiation, and thus de novo methylation, for the non-CG context.

### Chromatin state and methylation divergence

DNA methylation depends not only on DNA sequence features but also on the local state of chromatin. Prominent among the determinants of chromatin state are the location of nucleosomes and the combinations and modifications of their constituent histone proteins. These chromatin marks can interfere with higher-order organization that generates proximity in three dimensions between regions that are distant in the genome. In concert with other regulatory proteins, all of these chromatin marks bring about a compaction or relaxation in certain segments of the genome that supports or counteracts the silencing of genes. In this section, we want to investigate whether regions that have a different chromatin state also differ with respect to methylation divergence.

#### Inaccessible chromatin accumulates methylation polymorphisms

The distinction between eu- and heterochromatin is not sufficient to characterize the variation of chromatin states present in the *Arabidopsis thaliana* genome. In order to compare methylation divergence with annotated chromatin features, we used a comprehensive classification of regions into chromatin states based on a multitude of chromatin marks. Sequeira-Mendes et al. [31] have identified nine different chromatin states based on DNA methylation, nucleosome occupancy, presence of different histone variants and modifications, and transcriptional activity.

**Fig. 4:**
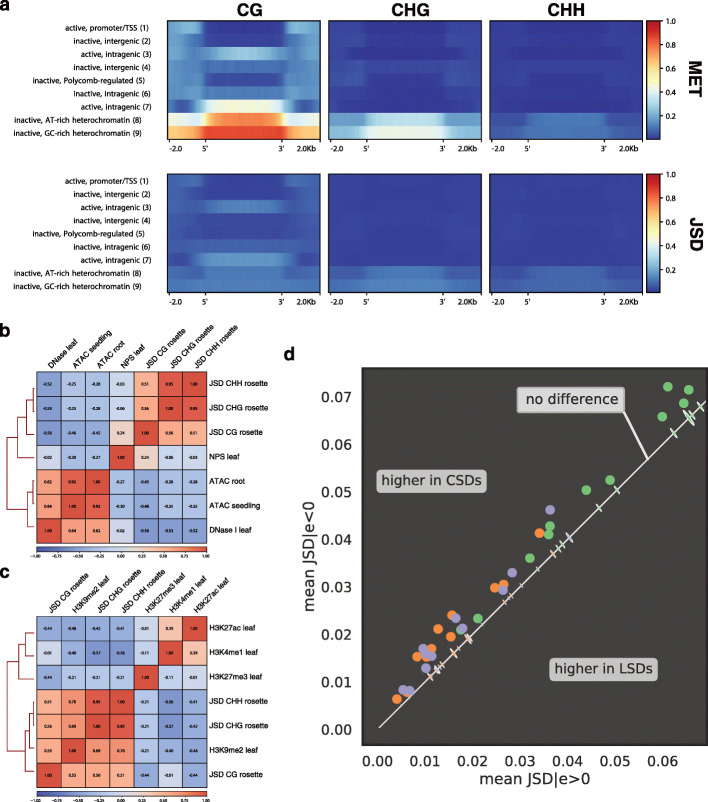
The influence of chromatin state on methylation divergence. **a** MET (top) and JSD (bottom) profiles over regions defined by different chromatin states for rosettes. The profile comprises 2-kb flanks and color-codes the arithmetic mean in non-overlapping, 50-bp bins. **b** Hierarchical clustering of JSD (rosette) with chromatin accessibility signals using Spearman’s *ρ* over non-overlapping, 50-kb intervals. Wherever applicable, replicate signals have been averaged. **c** Hierarchical clustering of JSD (rosette) with histone marks. The H3K9me2 signal is log2(H3K9me2/H3) as in [32]. **d** Mean JSD in regions characterized by positive (*x*-axis) and negative (*y*-axis) HiC-eigenvalues (*e*). The dots indicate the observed pair of values for different C contexts (color-coded) and all sources (not annotated). The kernel density estimates (small ellipses) represent the corresponding random distributions for reshuffled genomic bins. The dashed bisecting line divides the plane into regions of higher JSD in compacted (CSDs) and loose (LSDs) structural domains as defined in [33]

Figure 4a summarizes the profiles of the arithmetic mean of MET and JSD across regions (and 2 kb flanks up- and downstream) distinguished by chromatin state for the rosette. The findings for rosette leaves illustrate by and large a general pattern but some deviations are observed for the heterochromatic states in certain reproductive tissues, e.g., the vegetative cell of pollen and the endosperm of seeds (Additional file 1: Fig. S3). Here, we focus on the rosette profiles in the CG context since only the heterochromatic states 8 and 9 show a modest increase in MET and JSD in the non-CG context.

As expected, MET levels are highest in the heterochromatic states 8 and 9, followed by the transcriptionally active, intragenic states 3 and 7 and the inactive states 4 (intergenic) and 6 (intragenic). MET and JSD levels usually increase concomitantly (Fig. 4a), except for the intergenic state 4 where an increased MET is not mirrored by an increased JSD. The JSD levels in the active states 3 and 7 tend to be similar to those in the heterochromatic states while the inactive state 6 shows lower JSD. If we focus not only on the levels in a specific region but on the whole profile, including boundaries and flanking regions, we see further differences among the intragenic as well as the heterochromatic states with elevated JSD. In state 3, MET shows a small dip close to the boundaries, increasing again within the flanking regions, and JSD is slightly higher than in the flanks. In state 6, however, MET actually drops and JSD barely differs compared to the flanks. In contrast to both states 3 and 6, a clear upsurge in MET and JSD is observed for state 7. This state shows a MET profile similar to those of the heterochromatic states 8 and 9. However, for state 8 (AT-rich) JSD drops around its boundaries, which is not observed in state 9 (GC-rich).

According to Sequeira-Mendes et al. [31], the heterochromatic states have a high propensity for DNAse I-inaccessible sites. Their increased JSD suggests that inaccessible regions tend to harbor more methylation polymorphisms than accessible regions. This is confirmed by a negative correlation of JSD with DNAse I- [34] and ATAC-seq [35] signal levels (Fig. 4b, see Additional file 1: Fig. S4 for MET). In accordance, nucleosome positioning (NPS) shows a positive correlation with CG-JSD; however, the correlation is rather weak and even absent in the non-CG context. Another histone mark that is associated with heterochromatic states is H3K9me2 (Additional file 1: Fig. S8). This is the major silencing mark in plants and is involved in a positive feedback loop with DNA methylation to maintain genome stability [32]. Accordingly, this histone mark displays a strong positive correlation with methylation divergence (Fig. 4c). Accessibility also seems to affect the divergence of intragenic state 7. This state, which is usually located in gene bodies, shows the sharpest increase in JSD among the intragenic states and tends to have more inaccessible sites than other intragenic states. The increase of JSD in the states close to the coding sequence (3) and the transcription termination site (6) seem to be unrelated to accessibility; in fact, state 3 is the most accessible state. Hence, inaccessibility alone cannot explain the intragenic increases in JSD, and we have to consider how chromatin state is determined at the underlying level of histones.

#### Histone signatures characteristic for gene bodies and with depletion of activating marks are correlated with increased JSD

We have seen that states 3, 6, and 7 are the only intragenic chromatin states that show a noteworthy increase in MET and JSD in the CG context. It is therefore reasonable to investigate whether the differences between these states mirror distinct combinations of histone marks.

With respect to average levels of JSD in the different intragenic states, the highest levels are observed in state 7, followed by state 3, and then state 6, having the lowest levels. What are the differences between these intragenic states in terms of histone marks that could explain these differences? Let us first highlight common features. All three states are enriched in H3K4me1 and are depleted in H3K27me3. H3K4me1 is a chromatin mark typical for gene bodies. H3K27me3 is a *Polycomb* mark typical for intergenic, repressive chromatin enriched in chromatin states 2, 4, and 5. At genome scale (Fig. 4c), H3K4me1 shows a substantial negative correlation with JSD in the non-CG but not in the CG context. H3K27me3 shows the opposite pattern, a negative but rather weak correlation with JSD in the non-CG context but a substantial negative correlation with JSD in the CG context. These findings suggest that histone combinations characteristic for gene bodies are conducive to increased methylation divergence in the CG context. Gene bodies are also characterized by a lack of the histone variant H2A.Z, which accumulates close to the TSS [36]. Thus, we would expect increased methylation divergence with increased gene body likeness, that means lower H2A.Z levels. Indeed, for H2A.Z, the levels are largest in state 6, followed by state 3, equal to the genomic average, and smallest in state 7 [31]. This is the exact reverse of the order with respect to JSD, indicating that histone signatures typical for gene bodies correlate positively with CG-JSD.

Activating histone marks are enriched in state 3, followed by state 7, and finally 6 [31]. The lowest transcribed state 6 lacks the common activating marks H3K36me3 and H3K4me(2/3). The highly expressed but partially inaccessible state 7 does have high levels of the activating mark H3K36me3 but harbors only average H3K4me2 and even reduced H3K4me3 levels. Finally, the highly expressed, highly accessible state 3 contains all three activating marks. An activating histone modification that was not included in the chromatin state classification is H3K27ac, the antagonist to the *Polycomb* mark H3K27me3. It is mainly found in gene bodies and correlates positively with gene expression [37]. At genome scale, H3K27ac is negatively correlated with JSD in all three contexts (Fig. 4c).

In summary, our findings suggest that intragenic regions display increased methylation divergence if they have a histone signature typical for gene bodies and are depleted in activating histone marks.

#### Compacted structural domains show increased methylation divergence

The state of chromatin can also be defined based on the three-dimensional architecture of the chromosomes. In particular, Hi-C studies in *Arabidopsis thaliana* have revealed that the genome can be segmented into loose and compacted structural domains (LSDs and CSDs, respectively) [33]. LSDs show a high frequency of interactions with distal domains, while CSDs have a high frequency of local interactions. There is some evidence that CSDs represent a more repressive chromatin state.

We used the quantitative representation of this domain structure, the eigenvalues (*e*) associated with the principal component analysis of the Hi-C correlation matrix, to compare it against MET and JSD. While the magnitude of the eigenvalue does not have a biological meaning, its sign has been shown to indicate LSDs (*e*>0) and CSDs (*e*<0), respectively [33]. The sign of the eigenvalue has been identified previously in non-overlapping segments of 50 kb length. We have used this observed segmentation to quantify MET and JSD in LSDs and CSDs, respectively. Then we compared the average difference between these domains in the observed to that in randomly reshuffled segmentations (1000 random sign permutations) and computed an empirical estimate of the *p* value. In this case, the *p* value is defined as the probability to randomly obtain an average difference of the signals between CSDs and LSDs at least as extreme as the observed one, *P*(〈*S*_*e*<0_〉−〈*S*_*e*>0_〉>0∣*H*_0_), where *S* denotes the genomic signal (MET or JSD, respectively) and *H*_0_ is a random segmentation where the number of bins with positive and negative eigenvalues, respectively, is constrained to be the same as in the observed segmentation.

In line with the results obtained using the nine chromatin states, we found that both MET and JSD are increased in CSDs, corresponding largely to repressive chromosome domains (Fig. 4d and Additional file 1: Fig. S4b). Although the difference between CSDs and LSDs is small in magnitude, it is highly unlikely to be expected by chance; the empirical estimates of the *p* value are zero (Additional file 1: Table S3) and the distributions associated with the 1000 randomly reshuffled segmentations are very sharp and falling on the bisecting line in Fig. 4d that indicates no difference between LSDs and CSDs.

We conclude that compacted chromatin domains are prone to increased methylation and tolerate higher methylation divergence than loose chromatin domains.

### Methylation divergence of genomic features

In this section, our focus is on DNA methylation divergence in protein-coding genes and different TE categories. We want to analyze whether genes with high divergence have common functional or positional features, which could explain higher JSD in general and enrichment with metastable Cs (MSCs) in particular.

#### TEs are associated with increased methylation divergence

TEs are an important factor in sequence evolution as well as major targets of DNA methylation for the purpose of silencing [6, 38]. Silencing may not have accuracy at the single-base level, in which case we would expect TEs to display methylation polymorphisms.

**Fig. 5:**
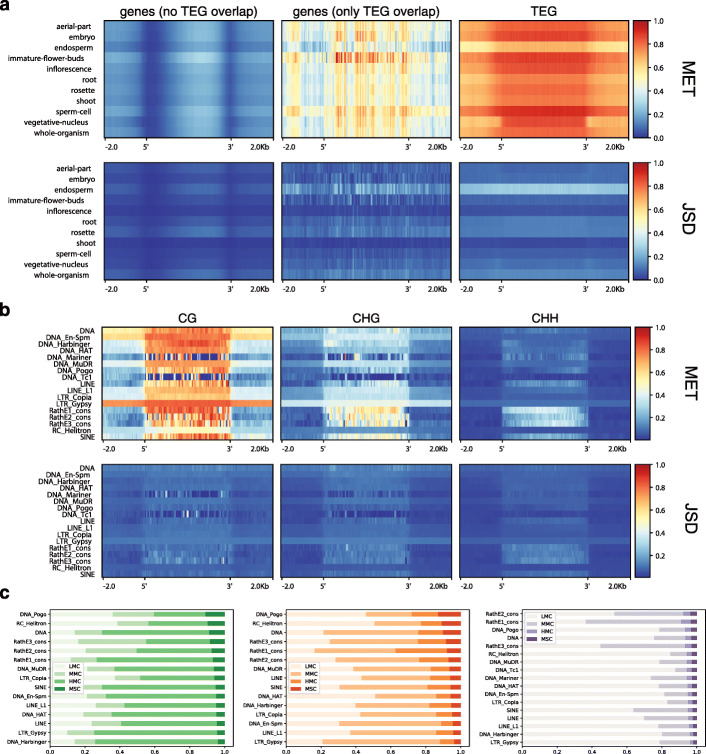
MET and JSD for genes and TEs. All metagene profiles use the arithmetic mean across genomic features with 2-kb flanks divided into 50-bp bins. **a** CG-MET and CG-JSD profiles in each source across non-TE genes without TEG overlap, non-TE genes with TEG overlap, and TEGs only. **b** MET and JSD profiles for rosette leaves in each context across TE superfamilies. **c** Proportion of C types for TE superfamilies in each C context sorted by MSC proportion

Figure 5a shows averaged MET and JSD profiles of all protein-coding genes. We have considered three mutually exclusive groups: (1) transposable-element genes (TEGs), as annotated by TAIR following [39] and used in the Araport 11 release [40], (2) genes that are not TEGs (non-TEGs) and have no overlap with TEG annotations, and (3) non-TEGs that overlap with TEGs.

Non-TEGs that do not overlap TEGs show the well-known gene body methylation profile for MET in the CG context, with the expected decrease in endosperm. The JSD profile largely follows the MET profile, although the typical shift of the methylation peak towards the 3 ^′^ end that is observed in metagene profiles is not apparent. The non-TEGs with TEG overlap have higher MET and JSD levels than the non-overlapping genes, but the TEGs themselves are highly methylated and also show increased JSD levels. Figure 5b shows the MET and JSD profiles of the rosette across all TE superfamilies in all three contexts. All TE superfamilies display high MET also with respect to their vicinity (DNA Mariner and DNA Tc1 are exceptions that may be due to noise as the coverage of these elements is low). Three superfamilies, DNA, DNA En-Spm, and LTR Gypsy, show increased MET also beyond feature boundaries and, thus, seem to fall into regions that are thoroughly methylated. Most of the TEs have an increased MET in the CHG context as well, but not in the CHH context. The exceptions are non-autonomous retroelements (SINE and RathE(1/2/3)_cons superfamilies) [39], showing increased MET also in the CHH context. The JSD profiles mainly follow the MET profiles. Figure 5c compares the proportions of C types among TE superfamilies in all three contexts, showing that RathE1, RathE2, and RathE3 have rather high proportions of MSCs and MMCs in all contexts, while DNA transposons (DNA, DNA Pogo, and RC Helitron superfamilies) show the highest proportions of MSCs in the CG and CHG context.

In summary, TEs are clearly associated with higher methylation divergence in genes. If the silencing of TEs does not require single-base precision, the increased single-site JSD in and around TEs makes sense. Note that a consistently methylated region can harbor polymorphisms at the single-base level and show high JSD, even though it was not identified as a differentially methylated region (DMR). This is apparently the case for TEs.

#### Metastable genes are associated with heterochromatic states

TEs are the main drivers of heterochromatin formation. As we have seen, heterochromatic states (chromatin states 8 and 9) have high levels of JSD. Here, we want to investigate whether protein-coding genes that have high proportions of metastable Cs are enriched in heterochromatic regions. Based on that, we will take a deeper look into the co-localization of these genes with conserved genomic elements that provoke heterochromatin formation.

In the following, we use the term metastable gene (MSG) for genes with a high proportion of MSCs. For each C context, we quantified the proportion of all C types in each gene, followed by sorting the genes according to the proportion of MSCs to select the top 5%. In some sources, this has lead to very low numbers of MSGs and we have excluded these from further analyses. To study the genomic distribution of MSGs without bias, we have normalized the MSG count by the total gene count in non-overlapping, genomic intervals. That is, a high signal in a genomic interval reflects a high proportion of MSGs.

**Fig. 6:**
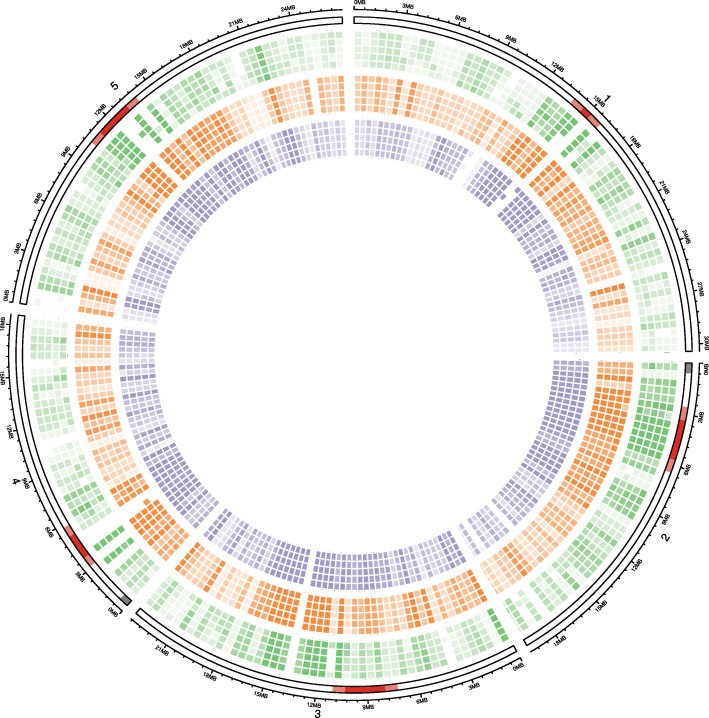
Genome-wide fraction of metastable genes. The axes from outside to inside are chromosome coordinates and idiograms highlighting centromeres (red), pericentromeres (light red), and telomeric heterochromatin (dark grey). The heatmaps show the fraction of genes that fall into the top 5% percentile of all protein-coding genes (excluding TE genes) with respect to the proportion of MSCs in the open reading frame. The colors encode C context following the colormap used throughout the paper, and the opacity encodes the fraction of metastable genes (MSGs). For each context, the order of sources from outside to inside is rosette, root, vegetative nucleus, sperm cell, and inflorescence

**Fig. 7:**
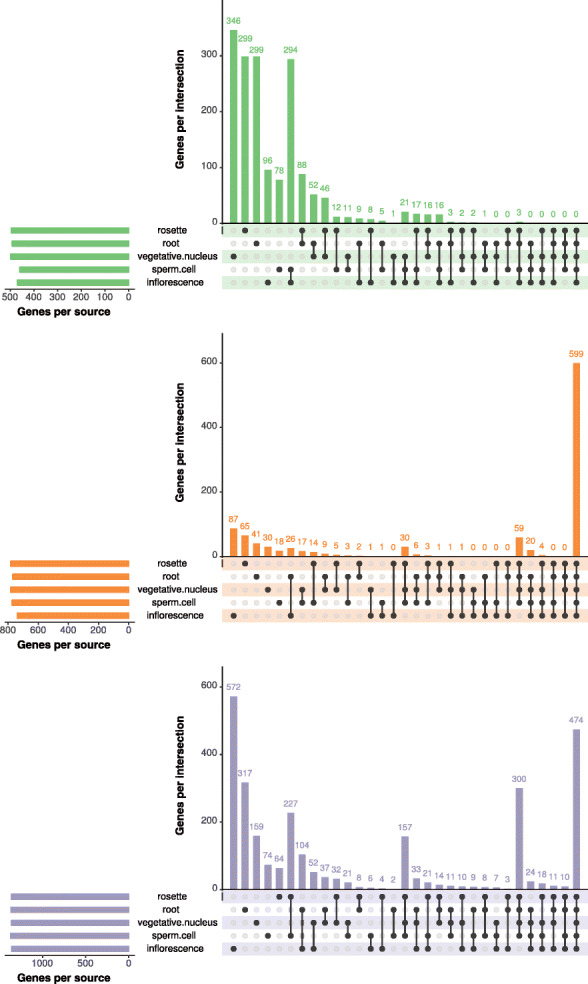
UpSet plots for MSGs by source in each context. The filled cells below the *x*-axis indicate the sources that are part of the intersection and the *y*-axis indicates the number of MSGs in the respective intersection. The total number of MSGs in each source is visualized by the bars on the left

Figure 6 shows the genomic distribution of this fraction of MSGs by context and source over 500-kb intervals. Most of the MSGs are enriched in pericentromeric and telomeric heterochromatin where we usually find a lot of TEs in *Arabidopsis thaliana*. However, there are also enrichments outside these regions in the chromosome arms. To see if the identified MSGs are unique, we have analyzed the overlap of MSGs by source and context. Figure 7 shows UpSet plots [41] for overlaps between sources for each context. Interestingly, the MSGs identified with respect to the CG context are mostly unique for each source. We see the opposite in the CHG context, where MSGs are, as a rule, shared among sources; a mixed picture emerges in the CHH context, where we find both unique and shared MSGs. For each source, the overlap among MSGs in different C contexts (Additional file 1: Fig. S5) shows that the majority of MSGs are unique for each context.

#### CMT2- and RdDM-targeted TEs correlate with increased methylation divergence in genes

The genomic overview plot in Fig. 6 hints at silenced heterochromatin as a determinant of methylation divergence in genes. Silencing often targets TEs to prevent their transposition and mutagenic effects. TEs can be classified into families and superfamilies [39] but also into elements that are targeted by different pathways of the DNA methylation machinery. Based on the analysis of mutants, two groups of TEs have been identified that show differential methylation if either CHROMOMETHYLASE2 (CMT2) or the RNA-dependent DNA methylation (RdDM) pathway is affected [20, 38, 42], referred to CMT2- and RdDM-targeted TEs hereafter. In this section, we want to explore whether MSGs preferentially co-localize with certain chromatin states and TE categories in order to highlight features that may trigger divergence at the level of DNA methylation.

**Fig. 8:**
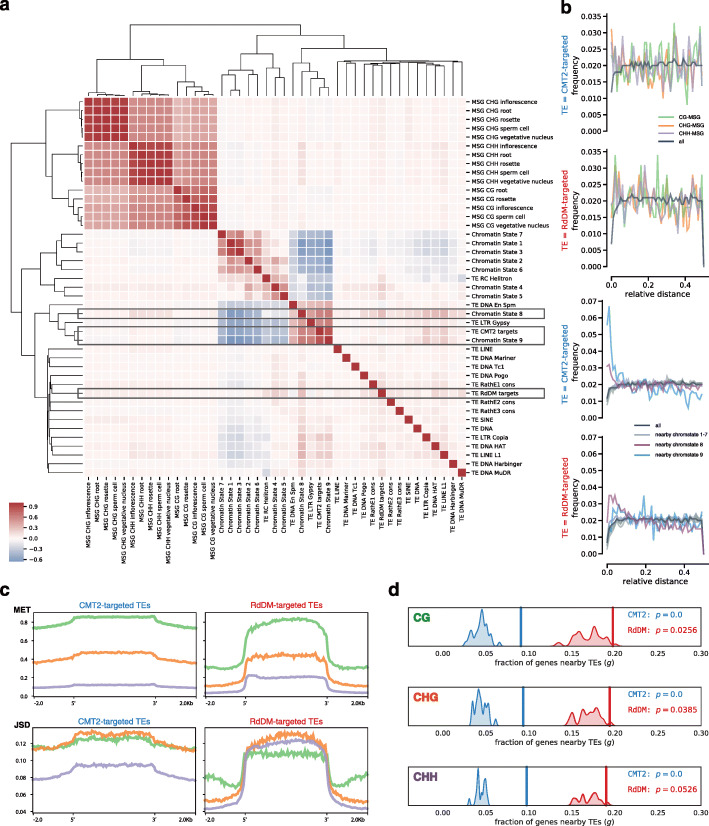
Spatial association of MSGs, TE categories, and chromatin states. **a** Hierarchical clustering for normalized enrichment scores of features based on Spearman’s *ρ* in non-overlapping, 50-kb intervals. **b** Spatial correlation measured by relative distance between, on the one hand, CMT2- and RdDM-targeted TEs and, on the other hand, genes that are metastable (left) and nearby certain chromatin states (right). **c** MET and JSD profiles for rosette leaves in each context across CMT2- and RdDM-targeted TEs. **d** Probability of metastable genes (MSGs) nearby CMT2- and RdDM-targeted TEs. The *x*-axis gives the gene count normalized to the total number of MSGs (top 5%, here for rosettes). The vertical lines show the observed fraction of MSGs nearby the targeted TE category. The kernel density estimates show the distribution of the fraction of genes nearby TEs for 10,000 randomly selected gene sets, each of a size equal to the total number of MSGs. The *p* value that is estimated by the randomization test is defined by *p*=*P*(*g*≥*g*_observed_∣*H*_0_), where the null hypothesis *H*_0_ is random selection of protein-coding genes that are not TEGs

We looked at the correlation of TE superfamilies, CMT2- and RdDM-targeted TEs, and chromatin states with MSGs to quantify the strength of co-enrichment of these features. Figure 8a shows the hierarchical clustering of these elements based on enrichment in non-overlapping, 50-kb intervals. The enrichments are normalized to the count of all protein-coding genes for MSGs, the count of all TEs for the different categories of TEs, and to the length of the interval (here 50 kb) for the coverage with chromatin states, respectively. The distance matrix used for clustering contains the average of Spearman’s *ρ* between the enrichments in the intervals. In addition, we used a relative distance measure (Fig. 8b, [43, 44]) and a randomized permutation test (Fig. 8d) to infer if MSGs are unusually close to CMT2- and RdDM-targeted TEs or certain chromatin states.

According to the hierarchical clustering by enrichment scores (Fig. 8a), there is a clear separation of regions that harbor MSGs from regions that harbor chromatin states and TEs. MSGs cluster by CG and non-CG context as usual. The cluster without the MSGs roughly splits into chromatin states and TEs with some “impurities” in the chromatin state cluster: In the bigger sub-cluster, all euchromatic chromatin states (1–7) correlate positively with the RC Helitron superfamily. These TEs are known to be near genes [38]. The smaller sub-cluster, which correlates negatively with the bigger sub-cluster, shows that heterochromatic states 8 and 9 are enriched together with LTR retrotransposons of the Gypsy superfamily, DNA transposons of the EnSpm/CACTA superfamily, and CMT2-targeted TEs. CMT2 preferentially targets Gypsy elements (Additional file 1: Fig. S6) and EnSpm/CACTA elements are known to accumulate along with LTR retrotransposons at pericentromeres, knobs, and TE islands [38]. These heterochromatic chromatin states and TE superfamilies, as well as the CMT2-targeted TEs in general, show increased levels of H3K9me2 (Additional file 1: Fig. S8), which is positively correlated with JSD (Fig. 4c). The remaining TE superfamilies and RdDM-targeted TEs form a cluster with rather weak correlation, but RdDM-targeted TEs show some co-enrichment with AT-rich heterochromatin (state 8), which itself is co-enriched with MSGs in the CHH context.

Figure 8b shows that in the vicinity of CMT2- and RdDM-targeted TEs one will find more MSGs than any gene from the background. If there would be no spatial correlation, we would expect a uniform frequency [44]. The increase of the frequency close to small relative distances to the TEs suggests a spatial correlation between MSGs and the targeted TEs. The tendency to co-localize with genes near chromatin states 8 and 9 is even more pronounced (8b, bottom), but there are some interesting differences between these states: RdDM-targeted TEs are close to genes nearby chromatin state 8, whereas CMT2-targeted TEs are close to genes nearby chromatin state 9. Apart from sequence composition (AT-rich vs. GC-rich), state 8 also differs from state 9 by increased levels of the *Polycomb* mark H3K27me3 and its genomic location [31]: state 8 is located in the chromosome arms, interspersed with euchromatic but inactive regions in states 4 (noncoding, intergenic) and 5 (*Polycomb*-regulated), whereas state 9 is characteristic for pericentromeres and is rather interspersed with state 8 only.

**Fig. 9:**
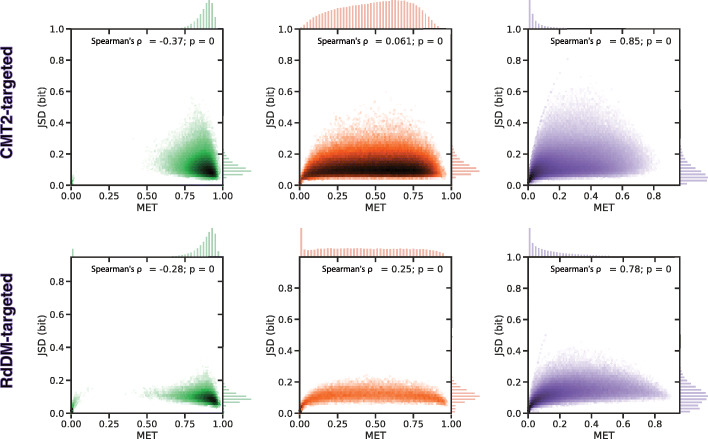
Context-specific MET-JSD phase planes for CMT2- and RdDM-targeted TEs. Methylome phase planes for rosette leaves. The margins display the distribution of MET and JSD, respectively. SciPy (scipy.stats.spearmanr) was used to compute Spearman’s *ρ* and the corresponding *p* value

The MET and JSD profiles of CMT2- and RdDM-targeted TEs clearly differ (Fig. 8c). It is not so much the level of these signals within the TE boundaries that differs, with MET being highest in the CG, intermediate in the CHG, and barely above noise in the CHH context for both TE categories. In contrast, both signals spread into the vicinity of CMT2- but not RdDM-targeted TEs, since RdDM’s role seems to be to “reinforce the boundary between TE and non-TE” [38]. It is also known that these pathways can target different parts of long TEs with the borders targeted by the RdDM and the middle targeted by the CMT pathway [45]. We have analyzed the MET and JSD profiles for long and short TEs (Additional file 1: Sec. S1.3 and Fig. S7) and see clear divergence peaks at the border of long TEs especially in the CHH context, which is known to be targeted in the wild type by RdDM [45]. The differences between long and short TEs are less pronounced in the CG and CHG context. However, it is known that RdDM is not exclusively targeted to the borders but that it can have an effect on the whole TE body [46]. The phase planes for the targeted TE groups (Fig. 9) are similar in the CG and CHH context with peaks at high and low MET, respectively; in the CHG context, MET is more evenly distributed but reveals a peak at LMCs in RdDM-targeted TEs that is absent in CMT2-targeted TEs.

It seems that metastable genes tend to be close to CMT2- and RdDM-targeted TEs. We tested for this by comparing the observed fraction of MSGs near TEs to the fraction in random gene sets (10,000 random draws of gene sets of the same size as the set of observed MSGs). As Fig. 8d clarifies, the distributions for random gene sets are consistently centered below the observed fractions (vertical lines), which makes it highly unlikely that the association of MSGs with CMT2- and RdDM-targeted TEs is by chance. Note that although the fractions for RdDM-targeted TEs are higher than those for CMT2-targeted TEs, the statistical evidence is even stronger for CMT2-targeted TEs.

In summary, these results suggest an important role for silenced, heterochromatic elements and associated TEs in driving intragenic methylation divergence. Notably, MSGs co-localize with CMT2- and RdDM-targeted TEs. Therefore, it is likely that TE insertions provoke imprecise de novo methylation through CMT2 and RdDM and thereby introduce single methylation polymorphisms that are stabilized through maintenance methylation and amplified into metastable (i.e., segregating) sites characterized by high JSD at the population level.

### Methylation divergence and gene expression

Location is not the only factor that affects gene methylation. In fact, a long-standing discussion in epigenetics concerns the interplay of DNA methylation and gene expression. Here, we are interested in the differences in DNA methylation (and methylation divergence) between differentially expressed genes.

To this end, we have profiled MET and JSD in two different categories of genes—the top 50 and bottom 50 genes by relative expression level. Since the expression of genes differs between tissues and organs, we have looked at these two categories in carpels, mature pollen, roots, and rosettes during the vegetative phase of the life cycle using data from the *Arabidopsis thaliana* expression angler [47]. Hence, different groups of genes were compared for each transcriptome source. We compared the metagene profiles across tissues, that is for a source-specific pair of gene sets (e.g., top and bottom expressed genes in rosette), we have looked for differences not only in the signals coming from the same source but also in the other sources that were included in this study. In addition, we performed a correlation analysis of all genes for available datasets (see Additional file 1: Sec. S1.4 and Fig. S9).

**Fig. 10:**
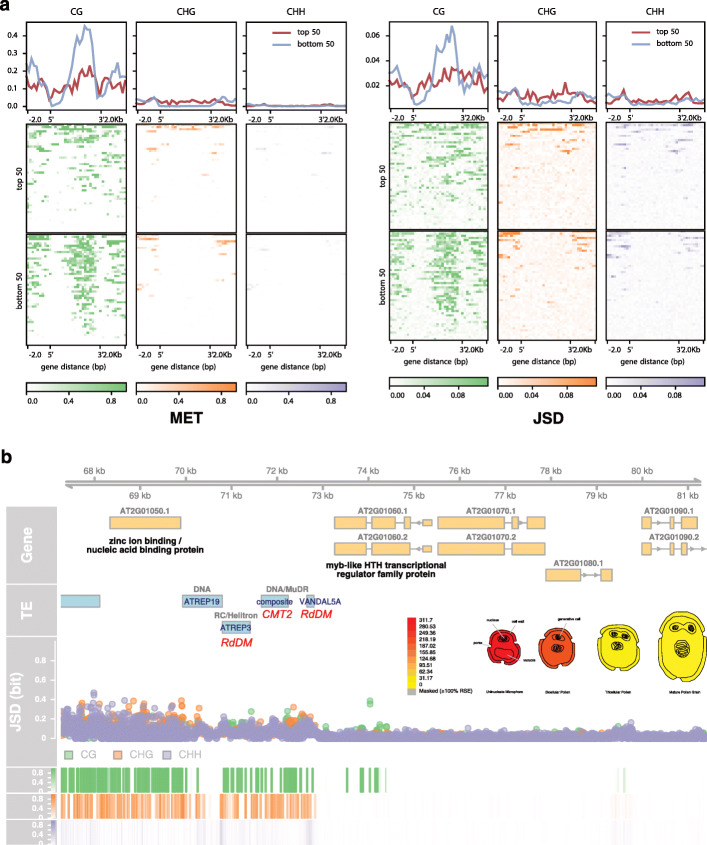
Differential DNA methylation for differentially expressed genes in mature pollen. **a** MET (left) and JSD (right) profiles (arithmetic mean, 2-kb flanks, 200-bp bins) from sperm cells for high- (top 50) and low-expressed (bottom 50) genes in mature pollen. The top panels show the metagene profiles over all top and bottom 50 genes, respectively. The bottom panels show profiles of each gene as a heatmap. **b** A genome browser view centered at a gene in the bottom 50 expressed group in mature pollen (myb-like HTH transcriptional regulator family protein; AT2G01060). The TE track highlights the TE family (blue), the superfamily (grey), and the targeting DNA methylation pathway (red) if applicable. The signal tracks are for JSD (scatter plot) and MET (heatmaps) in sperm cells using context-dependent colors as in the rest of the text. The eFP image shows relative gene expression of AT2G01060 and was generated with the “Tissue Specific Microgametogenesis eFP” of ePlant [48] using Affymetrix ATH1 array data [49]

While we observe a negative correlation between MET/JSD and gene expression in general (Additional file 1: Fig. S9), effectively, there is no difference in DNA methylation between the top 50 and bottom 50 expressed genes of all analyzed sources with one exception: in mature pollen, the gene bodies of downregulated genes show on average higher levels of MET and JSD in the CG context (Fig. 10a). The increased methylation divergence in these genes is not restricted to the sperm cell and vegetative nucleus methylomes, which make up the pollen methylome. It is also observed in methylomes from vegetative sources like rosette leaves. Interestingly, many of the genes that are repressed in mature pollen are expressed constitutively in the rest of the plant (see Additional file 1: Fig. S10 for a snapshot of the 9 least expressed genes). That means, although there is a difference in methylation between the top 50 and bottom 50 expressed genes in mature pollen, the methylation level of the bottom 50 genes themselves does not change in response to the repression during pollen development. One example is given in Fig. 10b. The *AT2G01060* gene, encoding a myb-like HTH transcriptional regulator family protein, shows gene body methylation and is upstream of CMT2- and RdDM-targeted TEs that are associated with high methylation in all three sequence contexts. The inset image shows the deactivation of this gene during pollen development.

In summary, our analysis of differentially expressed genes across many sources highlights numerous counterexamples to the general claim that variation in gene body methylation is somehow related to expression levels. The increased methylation in the bottom 50 genes expressed in mature pollen is probably a remnant of ancient methylation that is perpetuated through maintenance methylation: first, only CG methylation is different from the top 50 genes, and second, CG methylation does not change during development despite changes in gene expression. Taking also previous negative findings into account [20, 36], the significance of the correlation between gene expression and gene body methylation is still unclear.

## Discussion

This is, to our knowledge, the first application of JSD to DNA methylation or any epigenetic mark. As the symmetrical, smoothed version of the Kullback-Leibler divergence [23, 50], JSD is always well-defined and bounded. JSD and mutual information are closely related [51] and can measure any type of statistical dependence between variables [52], not being restricted to linear or monotonous relations. These and other unique properties of JSD have been discussed extensively in the literature [24, 53–57] and have led to successful applications in different fields [24, 58–60]. Interestingly, since JSD is based on the partitioning of a measure of variation, in this case the entropy of distributions, it embodies an idea that is also prevalent in the analysis of species [61, 62] and genetic diversity (*F*-statistics, [1, 63]). However, the extension of JSD to more levels of population subdivision, similar to hierarchical *F*-statistics [64], is outstanding. This would allow the apportionment of JSD to different groups or experimental factors and substantially increase the practical value of our approach.

Here, we have characterized the variation of organ-, tissue-, and cell-specific methylomes of *Arabidopsis thaliana* by applying JSD to methylation tables. These tables characterize each cytosine’s state by two counts, the number of methylated and unmethylated reads, which leads to contingency tables at each site. In this case, the weighted methylation level (MET) turns out to be a natural complement to JSD. Together, they define the state of each C-site with respect to the population, as illustrated in Fig. 1b. It is reassuring that MET has been identified previously as the most suitable summary statistic for the methylation level across many sites [65]. We want to emphasize here that methylation tables are by no means the only way to characterize the state of cytosine sites. For example, a methylation caller like Bis-SNP [66] would provide probability distributions over the sample space of “epigenotypes” *CC*,*MC*,*MM*, where C and M denote the unmethylated and methylated cytosine, respectively. The divergence of these distributions can also be analyzed with JSD by giving, for example, each distribution the same weight. In principle, any method or analysis pipeline that yields information in the form of a probability distribution can be used as the input for JSD, not only contingency tables. This makes JSD such a highly flexible framework to confront different types of data sets.

Different approaches already exist to detect differentially methylated cytosines (DMCs) and regions (DMRs) based on BS-seq data [67], some of them also based on the concept of entropy. However, we want to emphasize that it was not our aim to propose another approach to infer DMCs. The JSD statistic of single C-sites can be used to do hypothesis testing (see [24] and [68] on the relation between testing and information divergences) to identify DMCs. However, we do not advocate such a practice here because we do not want to know *if* a site differs with respect cytosine methylation but by *how much*. Moreover, DMCs and DMRs are usually restricted to case-control designs where two individuals or two groups are compared. The advantage of JSD for population studies is that it applies to many individuals or groups and that it acknowledges the intrinsic hierarchy in the data set by taking into account the variability at each site. In that sense, JSD is rather comparable to the analysis of variance (ANOVA) without assuming normally distributed variables and with the same variance (i.e., homoscedasticity). Regarding DMRs, it is important to realize that the average JSD of C-sites in a region does not equal the total JSD of the region. The average JSD, which has been analyzed in this study, ignores the variation of JSD within the region, something that a proper regional divergence measure should take into account. However, the proper weighting to quantify the regional JSD is still an open problem. Thus, a region with increased average JSD of sites may indicate a robust DMR but can also just be a hotspot of divergent sites in the examined population.

One way to improve the present approach is to use better estimators of JSD and to also estimate the uncertainty of different estimators due to finite sampling from a population. The non-parametric “plug-in” estimator, which replaces probabilities by frequencies, is fast to compute and gives good results for large sample sizes. But better estimators exist for small sample sizes based on the *k*-nearest neighbor algorithm [69], and these may be relevant for estimating JSD at sites with small read depth. Also, the empirical estimator of methylation divergence is sensitive to uneven coverage at a site across the population—that is, if one or very few sampling units have a substantially higher read depth than the rest, their weights *π* will be close to one while the weights of all other units will be close to zero (see the definition of the weights in the “Methods” section). This leads to a bias towards lower values of JSD in the sample as compared to the population. On the other hand, the present estimator of JSD has minimum variance [24]. An improved estimator of population JSD should correct for the coverage bias while still having the minimum variance possible. For exploring divergent sites, at least, the bias towards false negatives rather than false positives is a tenet because selecting candidate sites for further study that turn out to be dead ends can be wasteful. Apart from developing and implementing better estimators for JSD, it is also useful to quantify their uncertainty. The uncertainty estimate can be in the form of (orthodox) confidence or (Bayesian) credible intervals. For example, if the sample size is small yet representative, resampling techniques like the bootstrap or the jackknife are appropriate to compute confidence intervals for estimators in the absence of exact formulae [70]. Alas, the determination of uncertainty with randomized algorithms is computationally expensive. A future task is to test if uncertainty quantification is feasible at the genome scale.

The divergence analysis of *Arabidopsis thaliana* underscores the remarkable overall stability of the methylome across different conditions and supports the conclusion that methylation patterns are to a significant extent determined by genome organization and not by environmental impacts [71, 72]. In regions that show a stable methylation state (i.e., hypo- or hypermethylated regions), JSD can uncover whether precision at the single nucleotide level is critical. If the region shows elevated JSD across the population, as is often the case in heterochromatin, the state of each single cytosine is less important than the state of the region itself. The consideration of different organs, tissues, and cell types highlights some features that may be overlooked if one focuses only on the whole organism or a single tissue: some loci are controlled by gene regulatory processes that unfold during development; in the non-CG context, mainly affected by de novo methylation, patterns of variation are determined by source rather than the difference between the CHG and CHH context. Thus, intra-individual data pooling can indeed obscure inter-individual differences. The same conclusion has been drawn recently in other plants [73, 74] and mammals [30, 75].

This study has shown that methylation divergence tends to be higher in the CG context compared to the non-CG context, especially in regions with gene body-like chromatin signatures. Although our analysis cannot rule out the influence of different Col-0 accessions, as there will surely be some genetic variation between plants maintained in different laboratories, we think that CG sites tend to display higher divergence because a methyl-group is occasionally lost during replication, and thus not maintained over subsequent cell divisions [27, 29]. These random perturbations can lead to differing methylation states in the daughter cells, which persist in the different lineages if there is no intrinsic tendency to reset the methylation state in the lineage that has lost methylation. This is a possible explanation for populations of cells and even whole plants with increased methylation divergence at CG sites, leading to metastable states at the population level [76].

Our results support and refine previous indications that TEs influence epigenomic variation [20, 77, 78]. *Arabidopsis thaliana* uses DNA methylation for the silencing of TEs using the RdDM and CMT2 pathways. The increased methylation divergence associated with these TEs indicates that the defense response is not precise at the single-cytosine level. That is, TEs seem to provoke an epigenetic layer of variation that may influence the “selective arena.” It is known that “selfish” genetic elements, although largely deleterious, can become functional [79, 80] and may even play a role in speciation [81]. Our results support the role of TEs as important drivers of evolution even if they are neutralized.

## Conclusions

We have implemented a fast, scalable method to perform genomic scans of divergence in large populations. This approach based on JSD is non-parametric; hence, it works without parameter tuning and model specification. JSD can be applied to any functional genomics data that maps a discrete probability distribution to a locus. Furthermore, its application is general and can be extended to analyze epigenetic variation between individuals, organs, tissues, or cells, including different cell lineages in heterogeneous tumors [8]. The application of JSD to methylome data in *Arabidopsis thaliana* shows that methylation divergence tends to increase the more closed, heterochromatic or silenced chromatin is. Our analysis emphasizes the dominant role of location for DNA methylation and its divergence, in particular the putative impact of nearby TEs that are targeted by CMT2 and the RdDM pathway.

## Methods

### Jensen-Shannon divergence

We chose JSD as a divergence measure mainly for two reasons: general applicability and flexibility. JSD is an information-theoretic divergence that can be applied to a set of probability distributions [23]. It is based on the concept of Shannon (or information) entropy [25] and can be interpreted in terms of the Kullback-Leibler divergence, as well as mutual information. As a divergence measure, it assigns a real number to a set of probability distributions with a common measure. This number reflects the variability of the set. In terms of Shannon entropy *H*, the general Jensen-Shannon divergence [55] for a set of distributions *P* is defined as

1 $$\begin{array}{*{20}l}  D (P) &= H(\sum_{j} \pi_{j} P_{j}) - \sum_{j} \pi_{j} H(P_{j}) \end{array} $$

2 $$\begin{array}{*{20}l} &= H \langle P \rangle - \langle H \rangle \end{array} $$

Here, the mixture distribution $ \langle P \rangle = \sum _{j} \pi _{j} P_{j} $ is the average of the probability distributions *P*_*j*_ with respect to the weights *π*_*j*_ and 〈*H*〉 is the corresponding average of the entropy of all *P*_*j*_. The weights are normalized, that is $ \sum _{j} \pi _{j} = 1 $ holds such that the mixture is a convex combination of distributions. Different weights can be assigned according to the importance of each distribution, which is useful for decision problems [23]. The Shannon entropy for a discrete distribution is defined as

3 $$  H(P_{j}) = - \sum_{k} P_{jk} \log_{b} P_{jk}.  $$

Here, *k*∈*Ω* is an event from the sample space with probability *P*_*jk*_, such that $ \sum _{k} P_{jk} = 1 $ is fulfilled. We follow the convention in information theory using the base *b*=2 to measure JSD in bit.

In terms of JSD, variation is equivalent to the expected loss of information upon “mixing” the data sources. Figure 1a illustrates this geometrically, using three binary distributions (i.e., with two events in the sample space). The maximum entropy in bit is log2(2)=1 bit in this case. The mixture entropy, *H*〈*P*〉, must lie on the red segment of the entropy graph, the exact location depending on the weights. Likewise, the point representing the corresponding average entropy, 〈*H*〉, of the set must lie on the red triangle. Due to the shape of the entropy graph, the red segment will always be above the red triangle, which means that *H*〈*P*〉≥〈*H*〉. Essentially, this inequality expresses the expectation that mixing different sources of information tends to increase uncertainty or, equivalently, leads to a loss of information. Due to this inequality, JSD is always bounded by zero and the logarithm of the size of the sample space, 0≤*D*≤ log(|*Ω*|).

### Methylation divergence

To compute methylation divergence over a reference genome, we have to estimate JSD from a set of methylation tables, that is, from read counts for two different events over a collection of methylomes. Let *i*,*j*, and *k* be indices for cytosine position in the genome, methylome in the population sample, and methylation state, respectively. Without loss of generality, we let *k*=1 indicate the methylated state and *k*=2 the unmethylated state, such that *n*_*ijk*_ denotes the corresponding read count. Based on these read counts, a straightforward estimate of population JSD is obtained by replacing probabilities with sample frequencies; this leads to the so-called *plug-in* or *empirical* estimator of JSD at each position *i*:

4 $$\begin{array}{*{20}l}  {\widehat{D}}_{i} & = H(\sum_{j} {\widehat{\pi}}_{ij} {\widehat{P}}_{ij}) - \sum_{j} {\widehat{\pi}}_{ij} H({\widehat{P}}_{ij}) \end{array} $$

5 $$\begin{array}{*{20}l} & = H \langle \widehat{P}_{i} \rangle - \langle \widehat{H}_{i} \rangle \end{array} $$

Here, the distributions and weights are replaced by their empirical counterparts

6 $$\begin{array}{*{20}l} {\widehat{P}}_{ij} &= \frac{1}{n_{ij}} \cdot (n_{ij1}, n_{ij2},\ldots),~\text{and} \end{array} $$

7 $$\begin{array}{*{20}l} {\widehat{\pi}}_{ij} &= \frac{n_{ij}}{n_{i}}, \end{array} $$

where $n_{ij}=\sum _{k} n_{ijk}$ is the per-methylome coverage at position *i* in methylome *j* and $n_{i} = \sum _{j} n_{ij} $ is the total coverage of position *i*. That is, at each position, each sampling unit has a weight corresponding to its coverage relative to the whole sample. Grosse et al. [24] have shown that the plug-in estimator of JSD with data-dependent weights has some bias but that it is the maximum likelihood estimator of JSD with minimum variance. Table 2 exemplifies the computation of JSD at a single site using the plug-in, or empirical, estimator.

**Table 2:** How to compute the terms of JSD at site *i* for a sample of three methylomes. The result is ${\widehat {D}}_{i} = 0.58 - 0.39 = 0.19$. *NA* not applicable

	*n*_1_	*n*_2_	*μ*	*π*	*H*	*πH*
*P*_*i*1_	15	0	1.00	0.42	0.00	0.00
*P*_*i*2_	11	1	0.92	0.33	0.41	0.14
*P*_*i*3_	5	4	0.56	0.25	0.99	0.25
〈*P*_*i*_〉	31	5	0.86	NA	0.58	0.39

We have developed an open-source program in Python, tentatively called *Shannon* [82], with a simple command line interface to efficiently perform JSD scans for a large set of methylomes using the plug-in estimator, see Additional file 1: Sec. S1.1 for more details.

A by-product of computing JSD at a site *i* is the methylation level ${\widehat {\mu }}_{i}$, which is the weighted average of the methylation levels ${\widehat {\mu }}_{ij}$ of the sampling units in the population sample:

8 $$  {\widehat{\mu}}_{i} = \frac{ \sum_{j} n_{ij1} }{ n_{i} }.  $$

This is the plug-in estimate of the methylation bias (MET) within the population. Unless stated otherwise, we refer to the position-specific estimates $ {\widehat {D}}_{i} $ and ${\widehat {\mu }}_{i} $ whenever we speak of concrete JSD and MET values. Figure 1b shows that JSD and MET span a “phase plane” that visualizes the spectrum of methylation across the genome and the population. At the population level, each cytosine can be represented by a combination of JSD and MET, hence a point in the phase plane. Since *H*〈*P*〉 is an upper bound for JSD, all such points must fall within the region below the curve.

### Data preprocessing

To generate the metadata, custom Python scripts were used to query the European Nucleotide Archive (ENA) and NCBI’s Biosample database for all available *Arabidopsis thaliana* BS-seq runs which were subsequently curated semi-automatically to remove inconsistencies (see Additional file 2). The investigation was restricted to wild-type methylomes of the Col-0 accession and to tissues/organs (hereinafter called source), for which at least three methylomes were available, see the overview in Fig. 1c and the relevant metadata tables in the repository [83] (under results/tables/metadata).

Based on the metadata, methylation tables were generated by, first, mapping sequencing runs in fastq format (quality-filtered using TrimGalore!/cutadapt [84] and mapped to the reference genome (TAIR10) using Bismark [85]) and, second, making methylation calls with MethylDackel [86]. The methylation tables were subsequently indexed with tabix [87] to prepare the data for computing JSD with *Shannon* [82].

### Data analysis

For the downstream analysis, we used the current genome annotation Araport 11 [40]. The complete pipeline was implemented in Snakemake [88], mainly using the scientific python stack [89–92], R visualization libraries [41, 93, 94], and tools for genome analysis [43, 95, 96]. Further details are given in Additional file 1: Sec. S1.2. The complete pipeline and data are publicly available [83, 97].

## Supplementary information

**Additional file 1** Supplementary information. PDF file (supplement.pdf) with additional text, table references, and figures. **Section S1 Methods:S1.1***Shannon* — a command line app for computing JSD. **S1.2** Software environment for analysis. **S1.3** JSD/MET along TE bodies. **S1.4** Correlation between expression and JSD. **Section S2 Tables:Table S1.** Statistics for JSD and MET in all sources. **Table S2.** Mann-Whitney *U* test for differences in JSD between C contexts **Table S3.** Empirical *p*-values for getting at least the observed difference between CSDs and LSDs in 1,000 randomly reshuffled segmentations **Section S3 Figures:Figure S1.** Chromosome 1 tracks for the proportions of C types in all sources. **Figure S2.** Hierarchical clustering by source and C context using MET. **Figure S3.** Influence of chromatin state on methylation divergence. **Figure S4.** Correlation of chromatin accessibility and histone signals with MET **Figure S5.** Overlap of top 5% MSGs according by C context. **Figure S6.** TE superfamily composition of CMT2- and RdDM-targeted TEs. **Figure S7.** JSD and MET along transposable elements (TEs) and 2 kb flanking regions. **Figure S8.** H3K9me2 profiles over chromatin states and TE categories. **Figure S9.** Correlation between gene expression and MET/JSD. **Figure S10.** Tissue-specific expression of the bottom 50 genes of mature pollen.

**Additional file 2** metadata table. Excel file (metadata_Col-0_allGenotypes.xlsx) with all sequencing runs that form the basis for our analysis. A CSV version of Additional file 2 can be found in the Gitlab repository [83] at results/tables/metadata/metadata_handcurated_marc.csv.

**Additional file 3** Review history.

## Data Availability

*Shannon* is published under a MIT license in Gitlab [82]. The complete data analysis pipeline and filtered metadata files are available in a Gitlab repository [83]. A version of the pipeline along with all genomic data, tables, and figures is available under a CC BY 4.0 license in Zenodo [97].
