# Supplementary material for: Cell type-specific genome scans of DNA methylation divergence indicate an important role for transposable elements
Source: Genome Biol. 2020 Jul 13;21:172. doi: 10.1186/s13059-020-02068-2 (PMC7359245; doi:10.1186/s13059-020-02068-2)
Supplement: Supplementary file 1 — Additional file 1 Supplementary information. PDF file (supplement.pdf) with additional text, table references, and figures. Section S1 Methods:S1.1Shannon — a command line app for computing JSD. S1.2 Software environment for analysis. S1.3 JSD/MET along TE bodies. S1.4 Correlation between expression and JSD. Section S2 Tables:Table S1. Statistics for JSD and MET in all sources. Table S2. Mann-Whitney U test for differences in JSD between C contexts Table S3. Empirical p-values for getting at least the observed difference between CSDs and LSDs in 1,000 randomly reshuffled segmentations Section S3 Figures:Figure S1. Chromosome 1 tracks for the proportions of C types in all sources. Figure S2. Hierarchical clustering by source and C context using MET. Figure S3. Influence of chromatin state on methylation divergence. Figure S4. Correlation of chromatin accessibility and histone signals with MET Figure S5. Overlap of top 5% MSGs according by C context. Figure S6. TE superfamily composition of CMT2- and RdDM-targeted TEs. Figure S7. JSD and MET along transposable elements (TEs) and 2 kb flanking regions. Figure S8. H3K9me2 profiles over chromatin states and TE categories. Figure S9. Correlation between gene expression and MET/JSD. Figure S10. Tissue-specific expression of the bottom 50 genes of mature pollen. [file 13059_2020_2068_MOESM1_ESM.pdf]

METHOD

Supplement: Cell type-specific genome scans of DNA methylation divergence indicate an important role for transposable elements

Önder Kartal*, Marc W Schmid and Ueli Grossniklaus†

S1 Methods

S1.1 *Shannon* — a command line app for computing JSD

We are currently developing an open-source, command line application for POSIX-like operating systems. We chose Python for its general-purpose features and the availability of mature libraries for numerical computing, especially pandas and NumPy. The source code is available in GitLab [1]. The application needs two types of data inputs:

- tabix-indexed genome position files (GPFs) for each sampling unit in the population sample and
- a metadata table as a tab- or comma-delimited text file where each row corresponds to a sampling unit

The metadata table must have at least two columns: ID, to specify a unique name for each sample, and URL to specify the location of its GPF. In addition, the metadata can contain columns that further specify each sample (e.g. ecotype, tissue, cell type, etc.). The output is a BED-like file with JSD values for each genomic position. Below is the help message and a usage example for computing JSD at Chr1 where the count data are given in the 5th (mC, methylated base calls) and 6th column (C, unmethylated base calls) of the GPFs, respectively:

```
> shannon div -h
usage: shannon div [-h] [--prob]
                   [--chunk SIZE] -m FILE
                   -o FILE -s ID -c COLN
                   [COLN ...] -n NAME
                   [NAME ...]

JS Divergence for genome position files
(GPFs).

optional arguments:
  -h, --help            show this help
                        message and exit
  --prob                indicate that data
                        are probabilites
                        (default: counts)
  --chunk SIZE          set size of data to
                        process in-memory
                        (default: 10000)
                        - in terms of
                          expected number
                          of genome
                          positions
                        - higher numbers
                          lead to more
                          memory-hungry,
                          faster
                          computations

required arguments:
  -m FILE, --metadata FILE
                        metadata for GPFs
                        - comment lines (#)
                          are ignored
                        - values must be
                          comma- or tab-
                          separated
                        - first non-comment
                          line must be
                          header
                        - "url" and "label"
                          columns are
                          required
                        - if stdin is
                          metadata use
                          "--metadata -"
  -o FILE, --output FILE
                        output filepath
  -s ID, --sequence ID  query sequence
                        (chromosome/
                        scaffold) in GPF
  -c COLN [COLN ...], --dcols COLN
```

*Correspondence: oender.kartal@botinst.uzh.ch
Department of Plant and Microbial Biology & Zurich-Basel Plant Science Center, University of Zurich, Zollikerstrasse 107, 8008 Zurich, Switzerland
Full list of author information is available at the end of the article
†Correspondence: grossnik@botinst.uzh.ch

```
  [COLN ...]
                      column numbers
                      (1-based) in GPFs
                      that hold the data
  -n NAME [NAME ...], --dnames NAME
  [NAME ...]
                      names of data
                      columns following
                      the order in
                      --dcols

> shannon div -s Chr1 -c 5 6 -n mC C \
> -m meta.csv -o jsd_Chr1.bed
```

In order to efficiently handle a large number of GPFs, the program chunks the genome into regions and sequentially processes each region by merging data across all GPFs. Reading data relies heavily on fast random access to genomic regions using tabix and efficient collection of the GPF regions using Python generators to prevent I/O-bound slowdowns. Merging data relies on pandas's ability to concatenate differently indexed data frames and to handle missing values for certain combinations of sampling unit and position. To estimate JSD based on the merged count data, the computations on each chunk are done in-memory using NumPy arrays. As of now, we have only implemented the empirical ("plug-in") estimator of JSD. That is, we use the empirical distributions to calculate weights, mixtures and entropy terms.

S1.2 Software environment for analysis

Python *%watermark output*

```
CPython 3.5.6
IPython 6.5.0

snakemake 5.5.4
numpy 1.15.2
scipy 1.1.0
pandas 0.23.4
matplotlib 3.0.0
seaborn 0.9.0
altair 2.2.2
deeptools 3.3.0
pybedtools 0.8.0

compiler: GCC 7.3.0
system: Linux
release: 3.13.0-86-generic
machine: x86_64
processor: x86_64
CPU cores: 32
interpreter: 64bit
```

R *sessionInfo()*

- R version 3.5.2 (2018-12-20), `x86_64-pc-linux-gnu`
- Locale: `LC_CTYPE=en_US.UTF-8`, `LC_NUMERIC=C`, `LC_TIME=en_US.UTF-8`, `LC_COLLATE=en_US.UTF-8`, `LC_MONETARY=en_US.UTF-8`, `LC_MESSAGES=en_US.UTF-8`, `LC_PAPER=en_US.UTF-8`, `LC_NAME=C`, `LC_ADDRESS=C`, `LC_TELEPHONE=C`, `LC_MEASUREMENT=en_US.UTF-8`, `LC_IDENTIFICATION=C`
- Running under: `Ubuntu 14.04.4 LTS`
- Matrix products: default
- BLAS: `/usr/lib/libblas/libblas.so.3.0`
- LAPACK: `/usr/lib/lapack/liblapack.so.3.0`
- Base packages: base, datasets, graphics, grDevices, grid, methods, parallel, stats, stats4, utils
- Other packages: BiocGenerics 0.28.0, circlize 0.4.6, GenomeInfoDb 1.18.2, GenomicRanges 1.34.0, Gviz 1.26.5, IRanges 2.16.0, S4Vectors 0.20.1, UpSetR 1.3.3
- Loaded via a namespace (and not attached): acepack 1.4.1, AnnotationDbi 1.44.0, AnnotationFilter 1.6.0, assertthat 0.2.0, backports 1.1.3, base64enc 0.1-3, Biobase 2.42.0, BiocParallel 1.16.6, biomaRt 2.38.0, Biostrings 2.50.2, biovizBase 1.30.1, bit 1.1-14, bit64 0.9-7, bitops 1.0-6, blob 1.1.1, BSgenome 1.50.0, checkmate 1.9.1, cluster 2.0.7-1, colorspace 1.4-0, compiler 3.5.2, crayon 1.3.4, curl 3.3, data.table 1.12.0, DBI 1.0.0, DelayedArray 0.8.0, dichromat 2.0-0, digest 0.6.18, dplyr 0.8.0.1, ensembldb 2.6.6, foreign 0.8-71, Formula 1.2-3, GenomeInfoDbData 1.2.0, GenomicAlignments 1.18.1, GenomicFeatures 1.34.3, ggplot2 3.1.0, GlobalOptions 0.1.0, glue 1.3.0, gridExtra 2.3, gtable 0.2.0, Hmisc 4.2-0, hms 0.4.2, htmlTable 1.13.1, htmltools 0.3.6, htmlwidgets 1.3, httr 1.4.0, knitr 1.21, lattice 0.20-38, latticeExtra 0.6-28, lazyeval 0.2.1, magrittr 1.5, Matrix 1.2-15, matrixStats 0.54.0, memoise 1.1.0, munsell 0.5.0, nnet 7.3-12, pillar 1.3.1, pkgconfig 2.0.2, plyr 1.8.4, prettyunits 1.0.2, progress 1.2.0, ProtGenerics 1.14.0, purrr 0.3.0, R6 2.4.0, RColorBrewer 1.1-2, Rcpp 1.0.0, RCurl 1.95-4.11, rlang 0.3.1, rpart 4.1-13, Rsamtools 1.34.1, RSQLite 2.1.1, rstudioapi 0.9.0, rtracklayer 1.42.1, scales 1.0.0,

shape 1.4.4, splines 3.5.2, stringi 1.3.1, stringr 1.4.0, SummarizedExperiment 1.12.0, survival 2.43-3, tibble 2.0.1, tidyselect 0.2.5, tools 3.5.2, VariantAnnotation 1.28.11, xfun 0.5, XML 3.98-1.17, XVector 0.22.0, zlibbioc 1.28.0

S1.3 JSD/MET along TE bodies

JSD and MET along TE bodies were visualized with deepTools (version 3.3.0) [2]. TEs were split into a large (length $>$ 4 kb) and a small (1 kb $<$ length $<$ 4 kb) group. Average profiles were calculated with computeMatrix (options scale-regions –beforeRegionStartLength 2000 –regionBodyLength 5000 –afterRegionStartLength 2000 –skipZeros) and plotted with plotProfile.

S1.4 Correlation between expression and JSD

Arabidopsis ATH1 gene expression was obtained and processed as described [3]. Gene expression values of tissues matching to tissues used in this study were averaged (table available in the Gitlab repository [4] as samplesForExpressionVsMethylationOrJSD.csv in the results/tables/ subdirectory. Average JSD/MET values were then compared to gene expression values for a given context and tissue type.

S2 Tables

Table S1 Statistics for JSD and MET in all sources. See Zenodo repository [5] at `new/data/results/stats_Col-0_wt_[SOURCE].csv`

Table S2 Mann-Whitney U test for differences in JSD between C contexts. See Zenodo repository [5] at `new/data/results/tests-mannwhitneyu_jsd-between-context_Col-0_wt_[SOURCE].csv`

Table S3 Empirical p-values for getting at least the observed difference between CSDs and LSDs in 1,000 randomly reshuffled segmentations. See Zenodo repository [5] at `new/data/results/tab-pvalues_HiC-domains_mean.csv`

References

1. Kartal Ö, Schmid MW. Shannon - Measuring Genomic Diversity Using Information Theory. Gitlab; 2019. https://gitlab.com/okartal/shannon.
2. Ramírez F, Dündar F, Diehl S, Grüning BA, Manke T. deepTools: A Flexible Platform for Exploring Deep-Sequencing Data. Nucleic Acids Research. 2014 Jan;42(W1):W187–W191.
3. Gossmann TI, Schmid MW, Grossniklaus U, Schmid KJ. Selection-Driven Evolution of Sex-Biased Genes Is Consistent with Sexual Selection in *Arabidopsis thaliana*. Molecular Biology and Evolution. 2014 Mar;31(3):574–583.
4. Kartal Ö, Schmid MW. Meta-Methylome. Gitlab; 2019. https://gitlab.com/okartal/meta-methylome.
5. Kartal Ö, Schmid MW, Grossniklaus U. Data and Code for "Cell Type-Specific Genome Scans of DNA Methylation Diversity Indicate an Important Role for Transposable Elements". Zenodo; 2019.
6. Zhao S, Cheng L, Gao Y, Zhang B, Zheng X, Wang L, et al. Plant HP1 Protein ADCP1 Links Multivalent H3K9 Methylation Readout to Heterochromatin Formation. Cell Research. 2019 Jan;29(1):54–66.
7. Schmid M, Davison TS, Henz SR, Pape UJ, Demar M, Vingron M, et al. A Gene Expression Map of *Arabidopsis thaliana* Development. Nature Genetics. 2005 May;37(5):501–506.
8. Nakabayashi K, Okamoto M, Koshiba T, Kamiya Y, Nambara E. Genome-Wide Profiling of Stored mRNA in *Arabidopsis thaliana* Seed Germination: Epigenetic and Genetic Regulation of Transcription in Seed. The Plant Journal. 2005;41(5):697–709.

S3 Figures

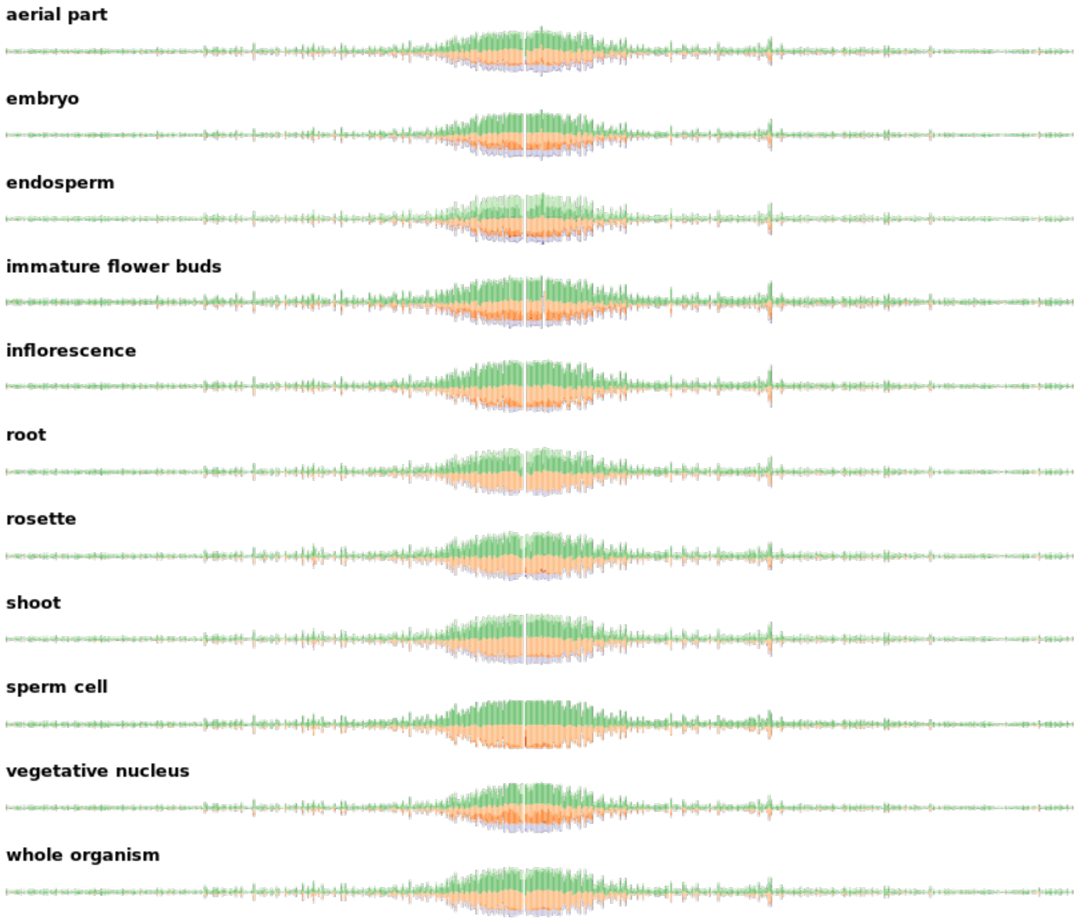

Figure S1 Chromosome 1 tracks for the proportions of C types in all sources. Proportions (stacked on y-axis, respectively) have been calculated at non-overlapping, 50 kb intervals (x-axis). Figures for all chromosomes can be found at `new/data/results/fig/plot-chromtrack-ctype_[CHROM].png`.

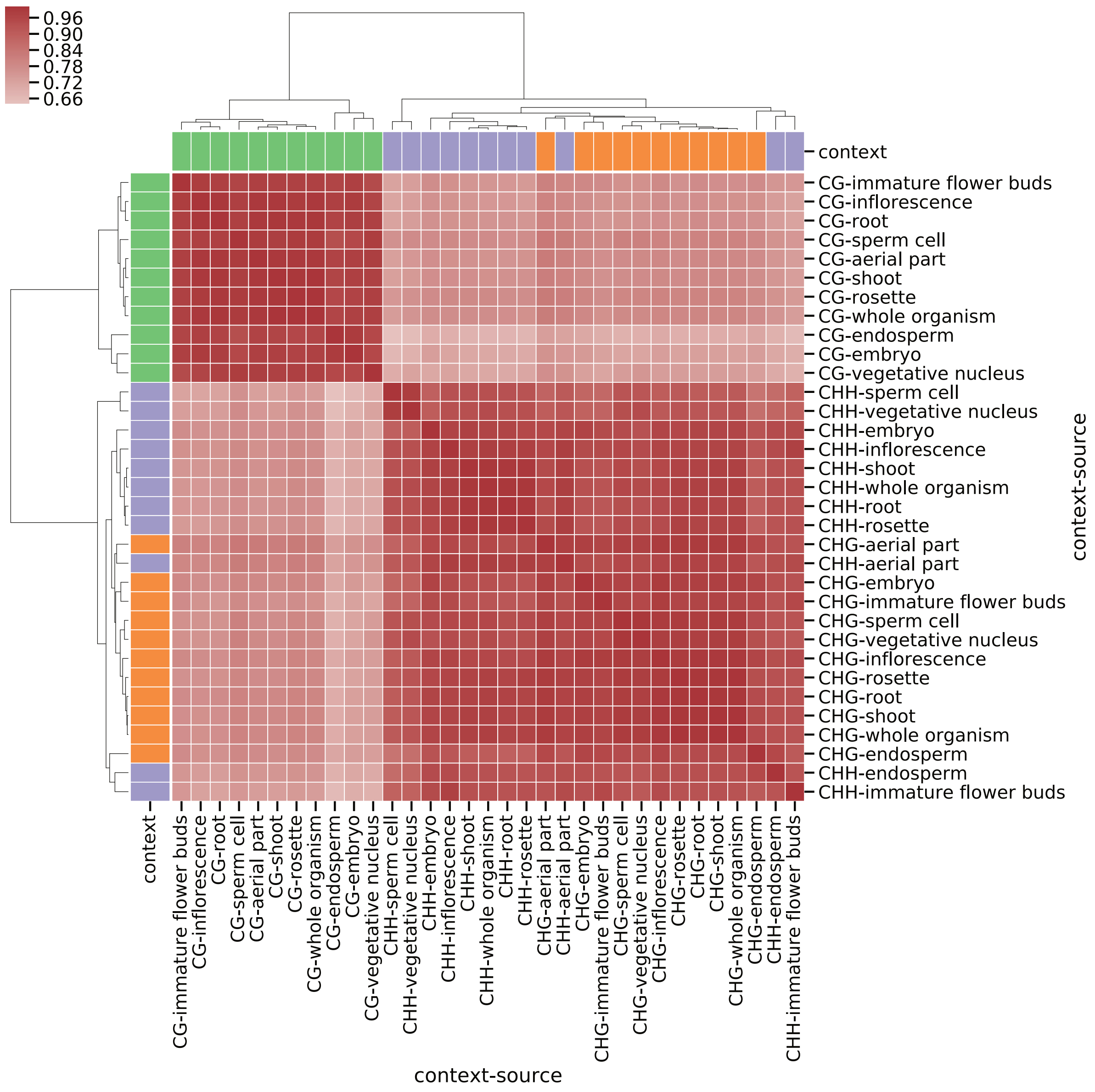

Figure S2 Hierarchical clustering by source and C context using MET. The distance matrix is given by the mean of Spearman's ρ at non-overlapping, 50 kb intervals over the whole genome.

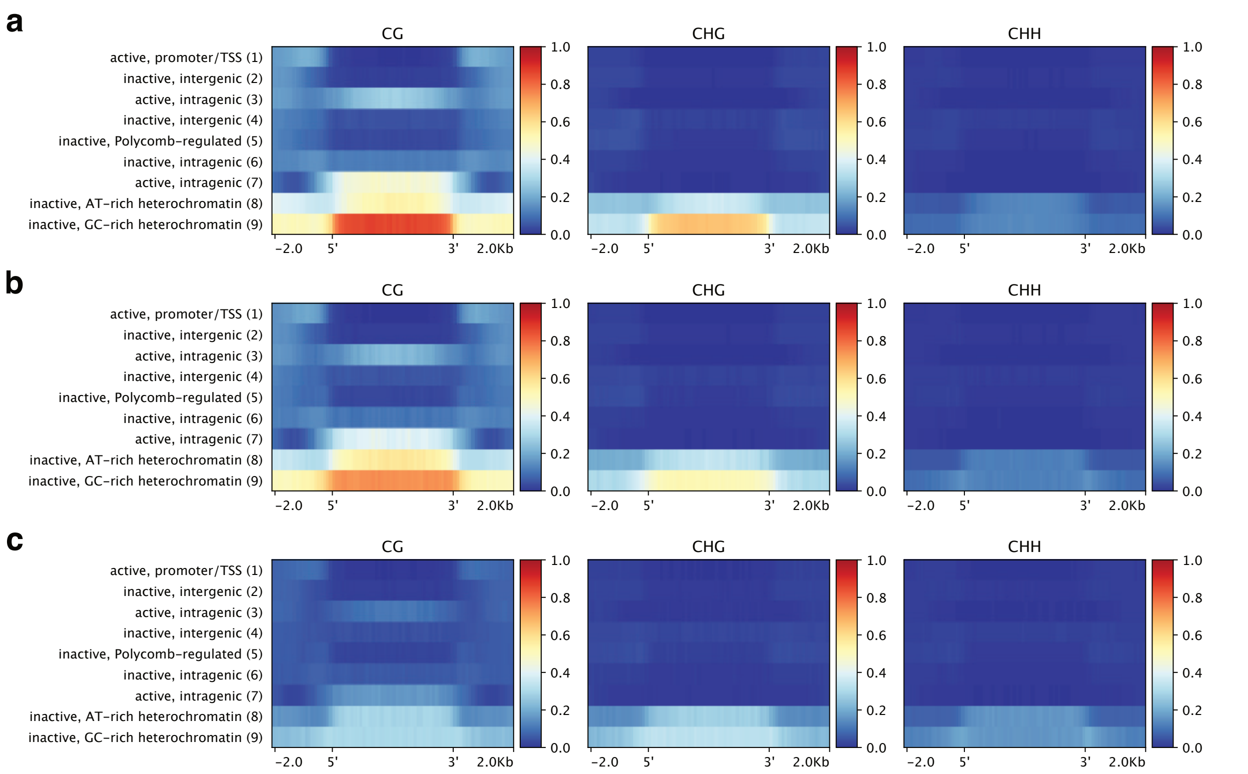

Figure S3 Influence of chromatin state on methylation divergence. The profile comprises 2 kb flanks and color-codes the arithmetic mean in non-overlapping, 50 bp bins (x-axis). **a** MET in vegetative nucleus. **b** MET in endosperm. **c** JSD in endosperm.

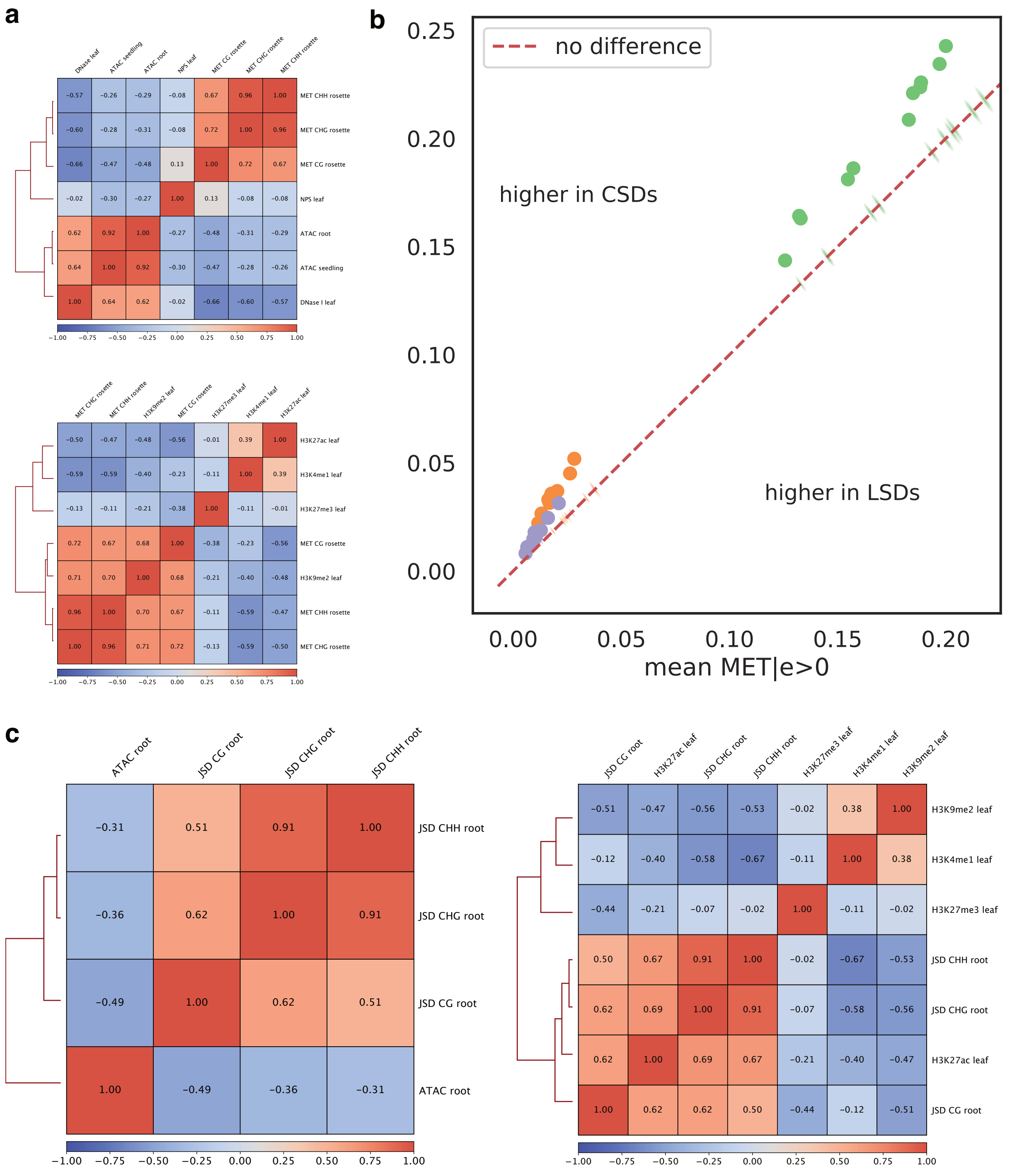

Figure S4 Correlation of chromatin accessibility and histone signals with MET. **a** Hierarchical clustering of MET (rosette) with chromatin accessibility signals (top) and histone marks (bottom) using Spearman's correlation coefficient over non-overlapping, 50 kb intervals. The number shows the Spearman's ρ averaged over non-overlapping 50 kb intervals. Wherever applicable, replicate signals have been averaged. The H3K9me2 signal is actually $\log_2(\mathrm{H3K9me2}/\mathrm{H3})$ as in [6]. **b** Mean MET in regions characterized by positive (x-axis, $\cdot|e>0$) and negative (y-axis, $\cdot|e<0$) HiC-eigenvalues, respectively. The dots show the observed pair of values for different contexts (color-coded) and all sources (not annotated). The kernel density estimates represent the distribution for eigenvalues of randomly reshuffled genomic bins. The dashed bisecting line divides the plane into regions where the score is higher in compacted (CSDs) or loose structural domains (LSDs), respectively. **c** Hierarchical clustering of root JSD with chromatin accessibility signals (left) and histone marks (right) using Spearman's ρ over non-overlapping, 50 kb intervals.

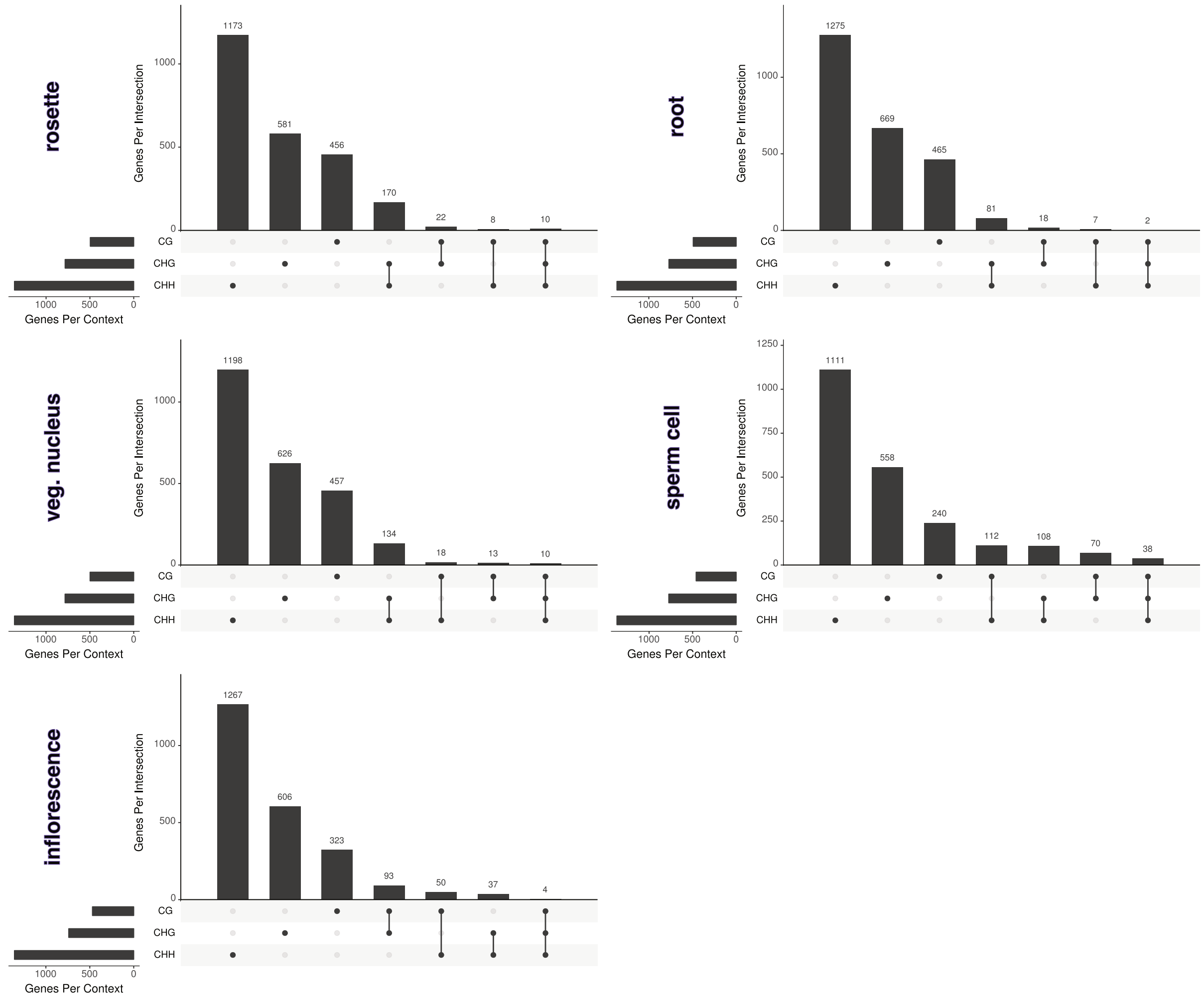

Figure S5 Overlap of top 5% MSGs according by C context. The filled cells below the x-axis indicate the C contexts that are part of the intersection and the y-axis indicates the number of MSGs in the respective intersection. The total number of MSGs in each context is given by the bars on the left, respectively.

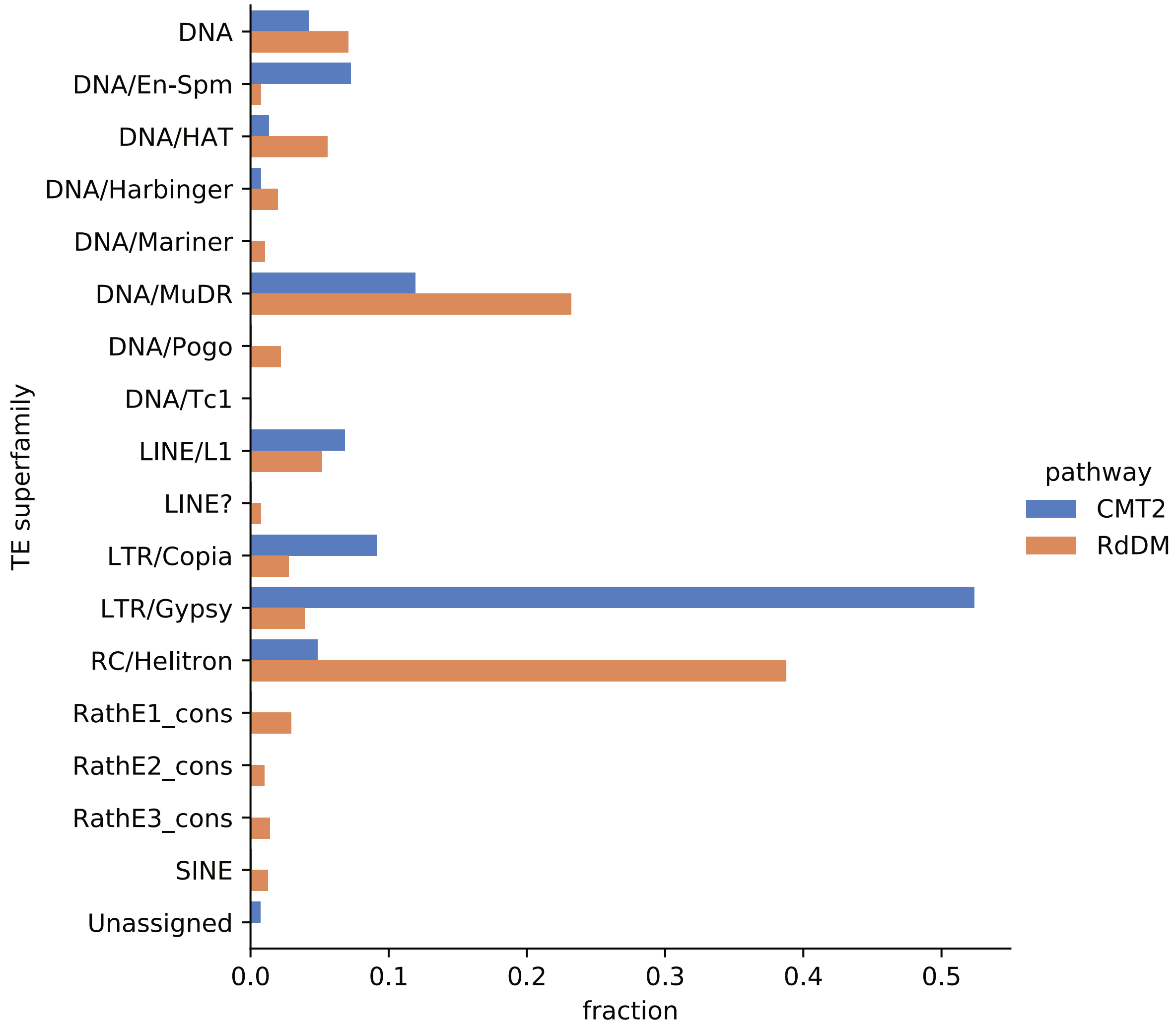

Figure S6 TE superfamily composition of CMT2- and RdDM-targeted TEs. The y-axis lists the TE superfamilies and the x-axis gives the fraction of TEs belonging to a given TE superfamily normalized to the total number of TEs in each DNA methylation category.

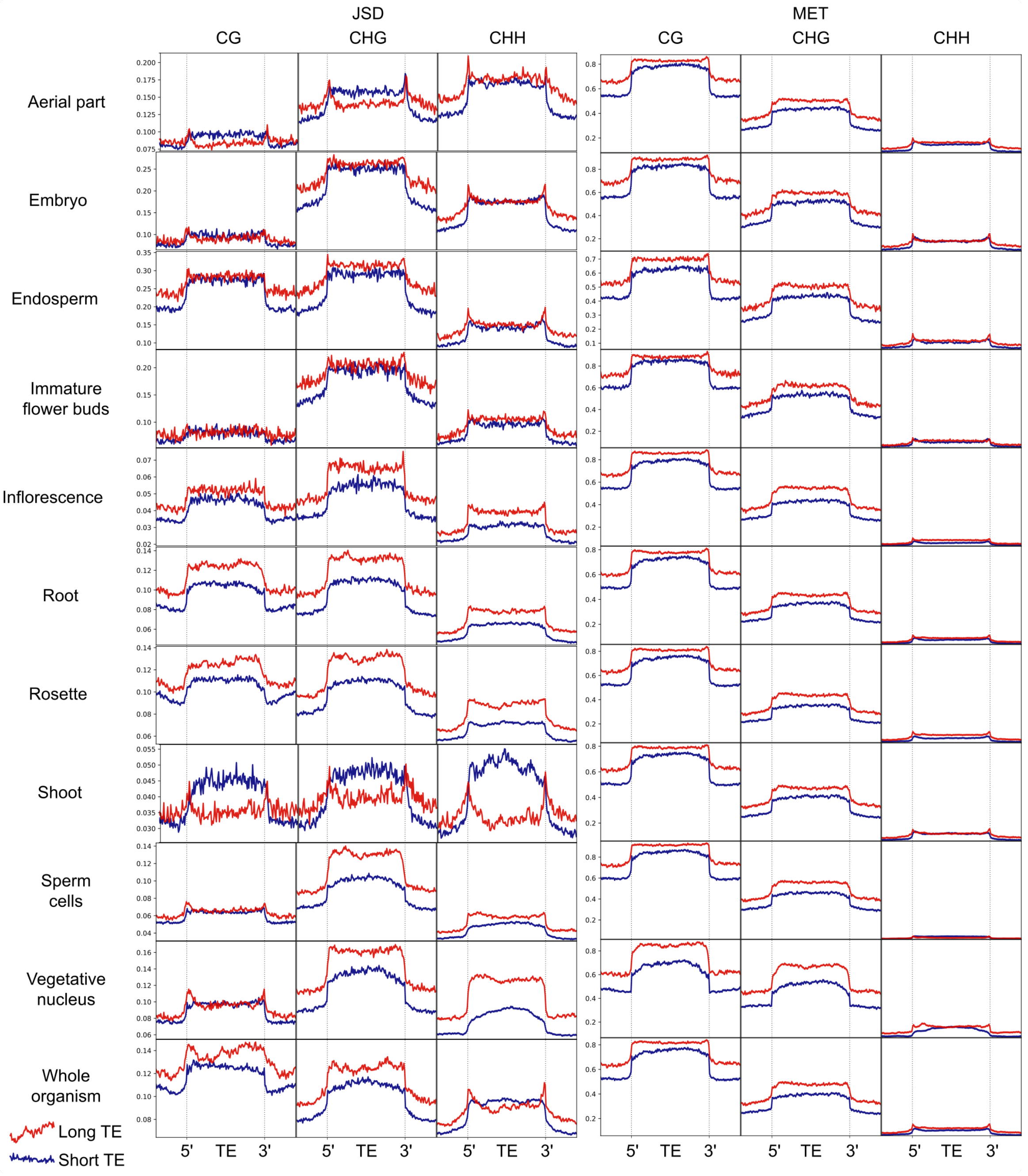

Figure S7 JSD (left) and MET (right) along transposable elements (TEs) and 2 kb flanking regions. TEs were split into two groups: long (length > 4 kb) and short (1 kb < length < 4 kb). In CHG context, JSD peaks at the borders of long TEs are visible in the arial parts and the shoot. In CHH, these peaks are in addition apparent in embryo, endosperm, inflorescence, and the whole organism. MET does not exhibit peaks at borders of long TEs in CHG context. However, MET peaks at borders of long TEs are visible in CHH context.

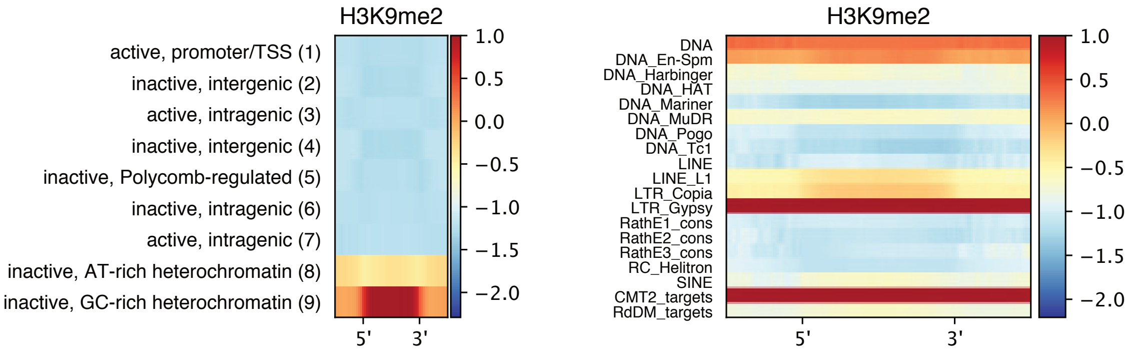

Figure S8 H3K9me2 profiles over chromatin states and TE categories. Each profile comprises 2 kb flanks and color-codes the arithmetic mean of the signal in non-overlapping, 50 bp bins (x-axis). The H3K9me2 signal is the log-transformed average of two replicates normalized to H3, $\log_2(\mathrm{H3K9me2/H3})$, as in [6].

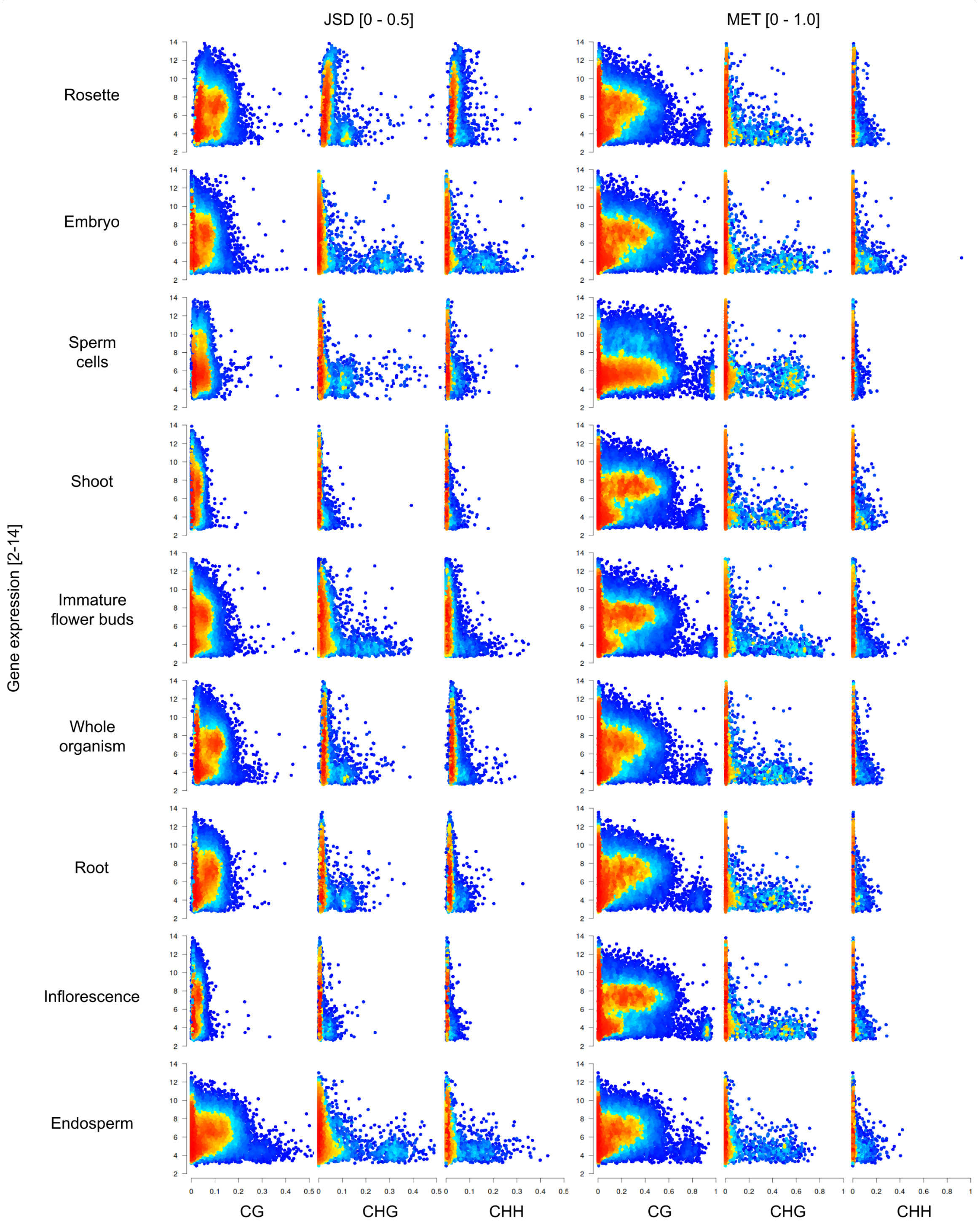

Figure S9 Correlation between gene expression and MET/JSD. Gene expression values (y-axis) compared to JSD (x-axis, left) and MET (x-axis, left) in all tissues for which data were available [3]. Gene expression values correspond to normalized and log2-transformed microarray signals (averaged over replicates and stages matching to the tissue). Values in brackets indicate the range of values shown in the plots. High JSD and MET in CHG and CHH context are generally associated with low expression values (< 8).

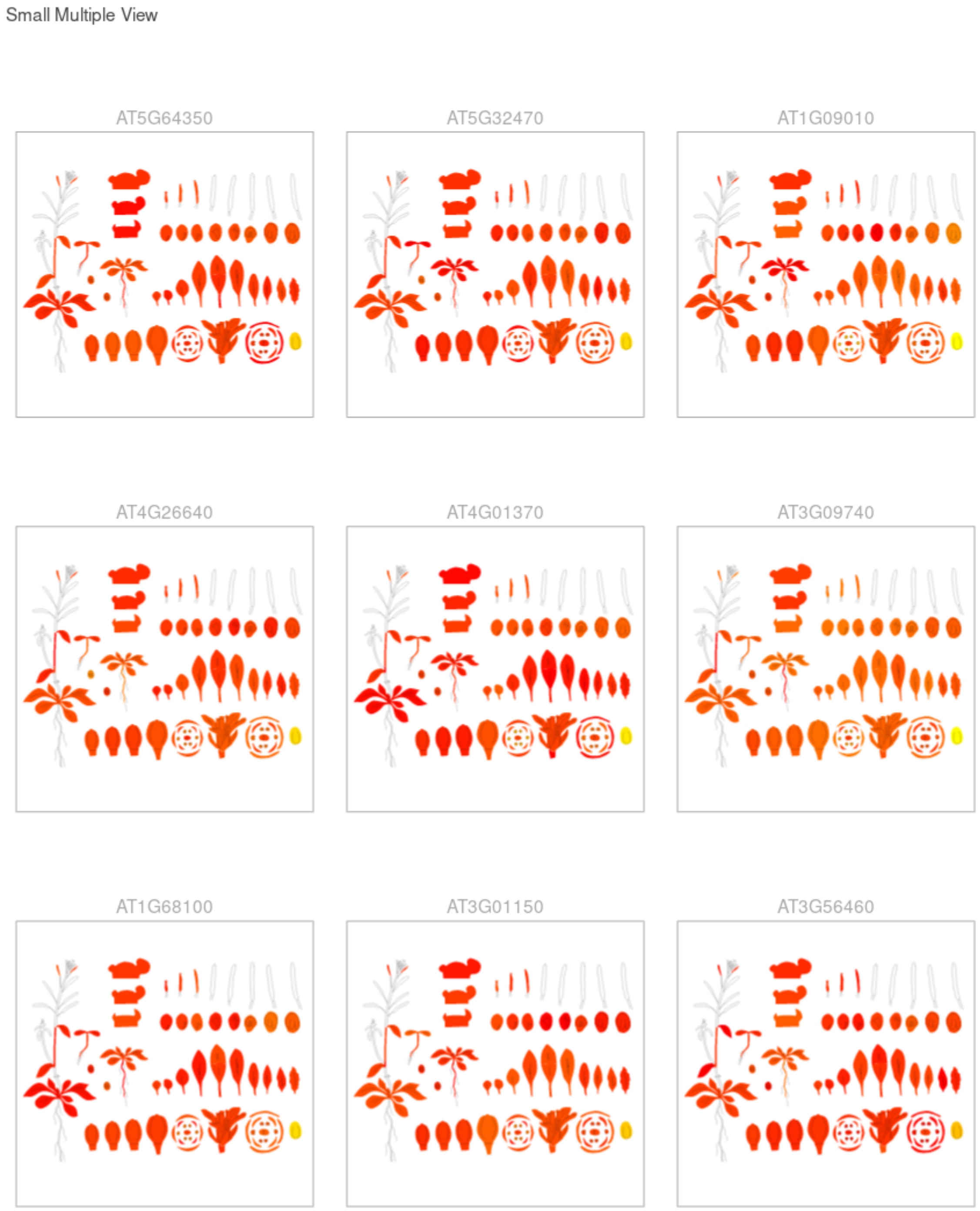

Figure S10 Tissue-specific expression of the bottom 50 genes of mature pollen. The small multiples view of ePlant shows the nine least expressed genes in the bottom 50 gene set of mature pollen. As described on the ePlant website, the gene expression data was "generated by the Affymetrix ATH1 array are normalized [*sic*] by the GCOS method, TGT value of 100." [7, 8]
