# Supplementary material for: Cell type-specific genome scans of DNA methylation divergence indicate an important role for transposable elements
Source: Genome Biol. 2020 Jul 13;21:172. doi: 10.1186/s13059-020-02068-2 (PMC7359245; doi:10.1186/s13059-020-02068-2)
Supplement: Supplementary file 3 — Additional file 3 Review history. [file 13059_2020_2068_MOESM3_ESM.docx]

Review History

**First round of review**

**Reviewer 1**

**Were you able to assess all statistics in the manuscript, including the appropriateness of statistical tests used? No.**

**Were you able to directly test the methods? No.**

**Comments to author:**

This manuscript assesses methylation diversity based on Jensen-Shannon divergence (JSD) across 482 methylome profiles from wild-type Col-0 ecotype. They measured JSD based on the tissue source of the methylomes and the sequence contexts, the two factors that are known to affect methylation diversity. They analyzed methylation diversity of distinct genomic features and evaluated the correlation of JSD-based methylation diversity with chromatin state and gene expression. Overall, the manuscript is well-written and many of the patterns found are well supported by published results.

One thing that bothers me is that the manuscript didn't demonstrate the application of JSD to identify regions that has been subject to selection, though it was mentioned many times in the manuscript, e.g. in both the Abstract and Introduction. JSD-based analysis of DNA methylation profiles across different Arabidopsis ecotypes may be a way to address this question. And these datasets in Arabidopsis is available (Kawakatsu et al., 2016, Cell, etc).

Another thing is that it is not clear how JSD is related to DMRs (Differentially Methylation Regions). Many published studies, including those in Arabidopsis, use DMRs to assess methylation diversity. What is the advantage of JSD compared with DMRs? It would be very interesting to connect the JSD levels with DMRs between accessions. Are regions with high JSD more likely to vary across ecotypes, and regions with low JSD less likely to vary across ecotypes?

Some other questions are as follows:
1, It is interesting that methylation diversity is higher in CG than in non-CG contexts. What is the possible reason and significance of this observation? Schmitz et al. (2011, Science) identified more CG-DMRs than non-CG DMRs among the Arabidopsis MA lines that are derived by single-seed descent for ~30 generations without selection. It is likely that the CG diversity may be caused by the different Col-0 accessions that are maintained by different labs.

2, Some regions may co-vary in all three sequence contexts (CG, CHG and CHH). Does the author observe a relationship between C-sites with high JSD for the three sequence contexts?

2, Lines 58-59 on the right panel of page 4, "The C-sites with low to moderate JSD are mainly LMCs", please cite the corresponding figure. What is the methylation level for C-sites with high JSD?

3, Lines 25-32 on the left panel of page 7, please cite the figures that illustrate "non-CG sites mainly occupy regions of lower JSD ". The sentence "First of all … majority of individuals." is confusing. Does the author mean "the non-CG sites with intermediate methylation levels (MET=0.5) have low JSD (Figure 2a), and thus, weak segregation of DNA methylation across populations; therefore, the 50% methylation is more likely to come from one of the two alleles in the diploid Arabidopsis genome?" There is another possible explanation, i.e., cell type heterogeneity. The tissues used to generate the methylome profiles contain many (different) cell types, so it is possible that the intermediate methylation levels are actually a combined methylation levels of many cells with either high/low methylation levels.

4, Lines 9-11 on the right panel of page 7, "The chromosomes … LMCs", it is hard to see enrichment of LMC from Figure 2d, please provide an zoom-in view of one chromosome arm as an example.

5, Figure 2c, the color for MSC is very unclear. It is hard to see an enrichment of metastable sites in heterochromain as suggested by lines 36-37 on the right panel of page 7.

6, Lines 40 on the right panel of page 8, change "DNAse1" to "DNase I".

7, Figure 3a is interesting, it suggests that high JSD tends to occur in intragenic regions (and heterochromatin to a lesser extent) for CG, but heterochromatic regions for non-CG contexts. Can the author provide a possible explanation for this? Figure 3b-3c, the JSD was from rosette while the chromatin accessibility data was from both leaf and root. It is possible that chromatin accessibility can vary across tissues, how could this affect the correlation between JSD and chromatin accessibility?

8, The author investigate the relationship between methylation diversity and genomic features, especially transposons, divided by transposon families as well as by the targeting pathways (RdDM or CMT2). It is known that the different parts of a transposon can be targeted by different pathways, especially for the transposons with large size, with the edges targeted by RdDM and the middle targeted by CMT2. Thus, it will be interesting to look at methylation diversity at different parts of large transposons.

9, Figure 5, it is hard to read the bar plots within the circles.

**Reviewer 2**

**Were you able to assess all statistics in the manuscript, including the appropriateness of statistical tests used? No.**

**Were you able to directly test the methods? No.**

**Comments to author:**

In "Cell-type specific genome scans of DNA methylation diversity indicate an important role for transposable elements", Kartal et. al. develop a non-parametric statistic to survey both the mean and variance in DNA methylation at single sites across the genome. The authors take advantage of the immense amount of bisulfite sequencing data from Col-0 to survey DNA methylation both within and across plant tissues. The authors show that although DNA methylation is remarkably stable within tissue (their focus was mostly on 300+ rosette samples), there were non-random enrichments of single-site DNA methylation variance, most notably in inaccessible chromatin. They performed a number of detailed analyses to dissect the relationship between genomic feature (gene, TE, etc) and both the mean and variance of DNA methylation in each. I've divided my concerns into major and minor comments below.

Major comments:
*     The authors invested in the development of an estimate of variance (called JSD) by borrowing from information theory. There was no mention of why this statistic is superior to other estimates of variance for data in contingency tables (i.e. variance of a hypergeometric distribution). Their conclusions rely on the development of this statistic and a discussion of its strengths would increase the novelty of their approach. A discussion, or supplementary figure, summarizing the effects of coverage on each statistic should also be included.
*     Throughout the manuscript the authors seek to interpret their summary statistics using population genetic theory. Their estimates of site-level DNA methylation mean and variance are certainly not synonymous with nucleotide diversity. The most common measures for nucleotide diversity is the average number of pairwise differences per site between individuals. Variance in the level of DNA methylation between samples (not even genetically distinct) cannot be interpreted using population genetic theory (i.e. it is not equivalent to standing genetic variation). I would suggest the authors remove all references to DNA "methylation diversity" and other references to population genetics throughout the text. As a next step it is reasonable to suggest a similar study using genetically diverse samples, but assumptions need to be carefully tested before an equivalency between DNA sequence polymorphism and DNA methylation polymorphism is drawn.
*     I have concerns about the sections of the manuscript that focus on regional differences between MET and JSD. In the introductory figures the authors detail the relationship between MET (mean) and JSD (variance) for single sites. A similarly careful approach to understanding the relationship between mean and variance for regions was not taken. Because mean and variance are often correlated, I'm concerned that their interpretations of changes of JSD for particular genomic regions is entirely dependent on the mean DNA methylation levels of these regions.
*     The authors did not explore the use of their statistic between tissue types. How does variance between tissue compare to variance within tissue?

Other comments:

*     Page 1, Column 1, Line 47. "The translation of these population level approaches to genome-wide chromatin marks is still missing, but the data available…" I believe there are missing reference here. Others have attempted to apply a population genetic framework to interpret sites with high mutability, like DNA methylation status (Charlesworth, Genetics, 2014; Wang, MBE, 2015; Van der Graaf, 2015, PNAS)
*     Page 2, Column 1, Line 26 "Due to its correlation with fitness relevant traits … soft inheritance". Please add citation.
*     Please explain how the empirical cutoffs for MSCs were defined in Fig 1B. Why was this determined by the vertex at MET 20/80% with H[P]?
*     It could be helpful to highlight MSC sites for each context in Fig 2A, either with a line, or colored points.
*     Page 2, Column 2, Line 58. "The C-sites with low to moderate JSD are mainly LMCs, as expected for the largely unmethylated genome of Arabidopsis". The figures are not referenced for many of the sentences interpreting the data and made it difficult to follow. This is 1 example. I think this should be referencing Fig 2B, but I don't see evidence for differences in JSDs.
*     Page 5, Column 1, Line 8 - "In fact, the joint distribution … HMC's". No figure cited here again.
*     Page 5, Column 1, Line 19- "genotype - and cell-type specific regulation play the main role in shaping the methylation landscape". No citation listed and the variation between genotypes was not analyzed in this paper.
*     Page 5, Column 1, Line 23 "Non-CG sites segregate within rather than among individuals" - No figures were cited in this section. Please reference the appropriate data.
*     Page 5, Column 1, Line 28. "First of all, a low JSD for a given site means that there is weak segregation across the population. That is these sites show intermediate methylation levels in the majority of individuals". The term "segregation" is not appropriate for tissue variation in methylation levels. Additionally, this entire section is speculative as the inference of methylation on only the maternal or paternal copies is not supported. Strand specificity or unbiased sampling at these sites compared to the genome-wide background was not determined. This section should be removed, or data added to support the proposed interpretation.
*     Fig 2E - Please explain chromosome track colors and or axes. Why is CG plotted on the positive y-axis and non-CG plotted on the negative y-axis. Why are CHG/CHH stacked? Does this include MSC?
*     Page 6, Column 1, Line 42 - "we ask how much diversity in DNA methylation certain regions can tolerate without their chromatin state being affected". Cause/effect relationships between chromatin status and DNA methylation cannot be tested with this data set.
*     Fig 3A - It is stated that MET and JSD are correlated, but subtle differences were discussed. Were there any statistical tests done to determine if difference existed?
*     Page 8, Column 1, Line 13 -" With respect to the levels of JSD … we observe the order S7 > S3 > S6)". Are these values the average across the regions? Was a statistical test done to determine difference?
*     Figure 3C. H3K9me2 is the most correlated with JSD, but not discussed in the text as much as the other marks.
*     Page 9, Column 1, Row 13 - "TEs can provoke higher methylation diversity". This title again implies causality, but causality cannot be determined.
*     Please define TEG more carefully. Does this refer to GAG/POL etc?
*     Page 9, Column 1, Row 30 - "The JSD profile … although the asymmetric bell shape is less pronounced". The bell pattern is not apparent in Fig 4.
*     Page 9, Column1, Row 59 - "In summary, TEs are clearly associated with higher methylation diversity in genes … in and around genes makes sense". I'm concerned that MET and JSD in regions are highly correlated.
*     Fig 6B - I could not follow the interpretation in the text for this figure and was not able to identify the relationship between MSG and TE type.
*     Section of gene expression differences: The authors analyze the 50 Top/bottom expressed genes for each tissue and then attempt to understand variation in gbM at these genes. In A. thaliana, gbM is typically enriched in moderately to moderately/highly expressed genes and then drops off for the most highly expressed genes in the sample. I'm concerned that their conclusions that there is a "dubious relationship" between gBM and expression is true for these 50 genes, but that these genes aren't representative of typically gbM genes.
*     Page 15, Column 2, Row 7 - "meta-methylome" term is referenced. I'm not convinced that a new term for studying mean and variance of DNA methylation is warranted here.
*     Methods - Please explain the weights for the JSD statistic more clearly in the methods.

**Authors Response**

**Point-by-point responses to the reviewers’ comments:**

First, we would like to thank the reviewers for their insightful and constructive criticisms. We have tried to address all the points they made in the revised version of our manuscript. Below, please find a point-by-point response to the reviewers’ comments. For convenience, we have numbered all comments consecutively, starting with Reviewer #1.

Reviewer #1

1. This manuscript assesses methylation diversity based on Jensen-Shannon divergence (JSD) across 482 methylome profiles from wild-type Col-0 ecotype. They measured JSD based on the tissue source of the methylomes and the sequence contexts, the two factors that are known to affect methylation diversity. They analyzed methylation diversity of distinct genomic features and evaluated the correlation of JSD-based methylation diversity with chromatin state and gene expression. Overall, the manuscript is well-written and many of the patterns found are well supported by published results.

We thank Reviewer #1 for this positive assessment of our manuscript.

2. One thing that bothers me is that the manuscript didn't demonstrate the application of JSD to identify regions that has been subject to selection, though it was mentioned many times in the manuscript, e.g. in both the Abstract and Introduction. JSD-based analysis of DNA methylation profiles across different Arabidopsis ecotypes may be a way to address this question. And these datasets in Arabidopsis is available (Kawakatsu et al., 2016, Cell, etc).

We agree with the reviewer that this would be a very interesting analysis to do. However, here we decided to analyze the diversity of different cell- and/or tissue types to demonstrate the usefulness of the method. Performing another analysis on a different data set without reference to different cell types would substantially change the focus of the manuscript and go beyond the scope of our current work. We therefore think that the analysis of selection is best left for the future. There is also a more fundamental reason why we think that scanning for selection with JSD alone requires caution and further developments. There are many covariates (also in the Kawakatsu et al. 2016, Cell, dataset) that make the identification of selection difficult because JSD, as of now, does not take these covariates into account. Also, it is in principle rarely possible to confirm with genome scans alone that the cause of a genomic signature is due to selection. For that, we often need a specific model that explicitly incorporates the effect of selection. Model-free (or rather model-agnostic) methods like JSD are appropriate for exploratory purposes and their results may serve as first approximations towards finding selected regions. We apologize if mentioning in the introduction that genome scans are often applied in population genomics to find selected regions has led to different expectations. Therefore, we tried to de-emphasize this aspect in the abstract as well as in the main text.

3. Another thing is that it is not clear how JSD is related to DMRs (Differentially Methylation Regions). Many published studies, including those in Arabidopsis, use DMRs to assess methylation diversity. What is the advantage of JSD compared with DMRs? It would be very interesting to connect the JSD levels with DMRs between accessions. Are regions with high JSD more likely to vary across ecotypes, and regions with low JSD less likely to vary across ecotypes?

We have reorganized the discussion section and added a paragraph to explain the difference between JSD and DMCs / DMRs. Regarding the question on different accessions, we refer to our response to comment 2: The Col-0 accession is the only one with a substantial amount of organ-, tissue-, and cell-specific methylation data, which was the main focus of our work. Introducing different accessions would also introduce more confounding genetic effects.

Some other questions are as follows:

4. [1,] It is interesting that methylation diversity is higher in CG than in non-CG contexts. What is the possible reason and significance of this observation? Schmitz et al. (2011, Science) identified more CG-DMRs than non-CG DMRs among the Arabidopsis MA lines that are derived by single-seed descent for ~30 generations without selection. It is likely that the CG diversity may be caused by the different Col-0 accessions that are maintained by different labs.

We thank Reviewer #1 for highlighting this possible explanation. We have amended and tried to clarify the paragraph in the discussion that highlights the role of the positive feedback mechanism of maintenance methylation for generating increased CG diversity. (“*This study has shown, that methylation divergence tends to be higher in the CG context compared to the non-CG context,…*”)

5. [2,] Some regions may co-vary in all three sequence contexts (CG, CHG and CHH). Does the author observe a relationship between C-sites with high JSD for the three sequence contexts?

We are not sure if we have understood the question here correctly but if the reviewer means exclusively the correlation between JSD signals in different contexts, we have only looked at the pairwise correlation coefficients in non-overlapping intervals over the entire genome as, for example, in Fig. 2e.

6. [2,] Lines 58-59 on the right panel of page 4, "The C-sites with low to moderate JSD are mainly LMCs", please cite the corresponding figure. What is the methylation level for C-sites with high JSD?

We have reformulated the whole paragraph (see also comment 21) to clarify the definition and rationale for C types. It is now the first paragraph in the subsection “*Divergence is higher in the CG than the non-CG context”.*

7. [3,] Lines 25-32 on the left panel of page 7, please cite the figures that illustrate "non-CG sites mainly occupy regions of lower JSD ". The sentence "First of all … majority of individuals." is confusing. Does the author mean "the non-CG sites with intermediate methylation levels (MET=0.5) have low JSD (Figure 2a), and thus, weak segregation of DNA methylation across populations; therefore, the 50% methylation is more likely to come from one of the two alleles in the diploid Arabidopsis genome?" There is another possible explanation, i.e., cell type heterogeneity. The tissues used to generate the methylome profiles contain many (different) cell types, so it is possible that the intermediate methylation levels are actually a combined methylation levels of many cells with either high/low methylation levels.

We have removed the entire subsection because it turns out to be rather confusing and speculative (see also comments 26 and 27). We have added a brief discussion of the intermediate MET levels to the previous subsection. We thank the Reviewer for highlighting this alternative explanation.

8. [4,] Lines 9-11 on the right panel of page 7, "The chromosomes … LMCs", it is hard to see enrichment of LMC from Figure 2d, please provide an zoom-in view of one chromosome arm as an example.

The plot is probably confusing because the LMCs are not shown. We have changed the legend and provide a zoom-in view. See also comment 28.

9. [5,] Figure 2c, the color for MSC is very unclear. It is hard to see an enrichment of metastable sites in heterochromain as suggested by lines 36-37 on the right panel of page 7.

We agree that the proportions of MSCs are hard to see. This is because, as detailed in the text and shown in Table S3, the genome-wide proportions of metastable sites are very low in all contexts with a maximum of 0.18% for the CG context. A change in color would not help much in this case. This is one of the cases where we think that Table S3 is actually more helpful than a bar plot. That is, we regard this plot only as an illustration. Therefore, we have added this table to the main text and address it in the corresponding paragraph (see also comment 23).

10. [6,] Lines 40 on the right panel of page 8, change "DNAse1" to "DNase I".

This has been corrected in the main text as well as in the Figures 3b and S5a.

11. [7,] Figure 3a is interesting, it suggests that high JSD tends to occur in intragenic regions (and heterochromatin to a lesser extent) for CG, but heterochromatic regions for non-CG contexts. Can the author provide a possible explanation for this? Figure 3b-3c, the JSD was from rosette while the chromatin accessibility data was from both leaf and root. It is possible that chromatin accessibility can vary across tissues, how could this affect the correlation between JSD and chromatin accessibility?

Regarding the increase of CG-JSD only in intragenic regions, we think that this is due to imprecise gene-body methylation that is mainly observed in the CG context. In the manuscript, we provided some support for the hypothesis that gene-body like signatures allow for greater divergence because the chromatin state may be unaffected by variation in single-site methylation. Heterochromatic regions are usually methylated in all three contexts but again, the increased single-site JSD indicates that fluctuations within these regions are common. Regarding the correlations between accessibility and JSD, it is indeed sometimes not easy to find out exactly which tissue or organ was sampled. We assumed here that there is not much difference between rosette and rosette leaf. We have added a subfigure to the supplemental figure on chromatin accessibility (Fig S4c) that shows the clustering for root JSD. The correlation with the root ATAC data is similar, but there is a different clustering when comparing root JSD signals with histone marks from leaves. This demonstrates what can be expected if the epigenetic state and gene activity differs between tissues.

12. [8,] The author investigate the relationship between methylation diversity and genomic features, especially transposons, divided by transposon families as well as by the targeting pathways (RdDM or CMT2). It is known that the different parts of a transposon can be targeted by different pathways, especially for the transposons with large size, with the edges targeted by RdDM and the middle targeted by CMT2. Thus, it will be interesting to look at methylation diversity at different parts of large transposons.

We thank the Reviewer for pointing out this interesting observation. We have performed the suggested analysis for short and long TEs and added the result in a supplementary figure: “*In CHG context, JSD peaks at the borders of long TEs are visible in the arial parts and the shoot. In CHH, these peaks are in addition apparent in embryo, endosperm, inflorescence, and the whole organism. MET does not exhibit peaks at borders of long TEs in CHG context. However, MET peaks at borders of long TEs are visible in CHH context.” We have added some sentences in the main text that refer to these findings.*

13. [9,] Figure 5, it is hard to read the bar plots within the circles.

We have separated the UpSet plots into a separate figure and increased their size.

## Reviewer #2

14. In "Cell-type specific genome scans of DNA methylation diversity indicate an important role for transposable elements", Kartal et. al. develop a non-parametric statistic to survey both the mean and variance in DNA methylation at single sites across the genome. The authors take advantage of the immense amount of bisulfite sequencing data from Col-0 to survey DNA methylation both within and across plant tissues. The authors show that although DNA methylation is remarkably stable within tissue (their focus was mostly on 300+ rosette samples), there were non-random enrichments of single-site DNA methylation variance, most notably in inaccessible chromatin. They performed a number of detailed analyses to dissect the relationship between genomic feature (gene, TE, etc) and both the mean and variance of DNA methylation in each. I've divided my concerns into major and minor comments below.

## Major comments

15. The authors invested in the development of an estimate of variance (called JSD) by borrowing from information theory. There was no mention of why this statistic is superior to other estimates of variance for data in contingency tables (i.e. variance of a hypergeometric distribution). Their conclusions rely on the development of this statistic and a discussion of its strengths would increase the novelty of their approach. A discussion, or supplementary figure, summarizing the effects of coverage on each statistic should also be included.

We have expanded and changed the Discussion part substantially to emphasize the difference between the variance of a distribution and JSD which applies to many distributions. We also discuss in more detail the effect of coverage on the weights in the JSD formula. When applied to contingency tables JSD is equivalent to the G test statistic (mutual information or likelihood ratio, see *Harremoës, P. (2014). Mutual information of contingency tables and related inequalities. ArXiv:1402.0092 [Cs, Math, Stat]*) but JSD, in contrast to the variance of the hypergeometric distribution makes no restrictive assumptions like fixed marginals. It is non-parametric so the counts could come from any distribution. We think that the assumption of fixed marginals also does not apply in this case as there is intrinsically no fixed number of reads that need to be distributed (without replacement) on the sites across the population.

16. Throughout the manuscript the authors seek to interpret their summary statistics using population genetic theory. Their estimates of site-level DNA methylation mean and variance are certainly not synonymous with nucleotide diversity. The most common measures for nucleotide diversity is the average number of pairwise differences per site between individuals. Variance in the level of DNA methylation between samples (not even genetically distinct) cannot be interpreted using population genetic theory (i.e. it is not equivalent to standing genetic variation). I would suggest the authors remove all references to DNA "methylation diversity" and other references to population genetics throughout the text. As a next step it is reasonable to suggest a similar study using genetically diverse samples, but assumptions need to be carefully tested before an equivalency between DNA sequence polymorphism and DNA methylation polymorphism is drawn.

We have replaced “methylation diversity” with “methylation divergence” in order to mitigate any confusion. We certainly did not want to leave the impression that 5mC is something like a 5^th^ base and JSD akin to nucleotide diversity. As we discuss now more thoroughly in the text JSD is a generic “diversity index” and one could also define a different methylation divergence not using contingency tables. However, we think it is appropriate to speak of a diversity index since one can interpret the exponential of JSD as the effective number of states at a site, following usage in ecology (Hill, Jost) where one defines diversity as the effective number of species.

*Hill, M.O. (1973). Diversity and evenness: a unifying notation and its consequences. Ecology 54, 427–432.*

*Jost, L. (2006). Entropy and diversity. Oikos 113, 363–375.*

17. I have concerns about the sections of the manuscript that focus on regional differences between MET and JSD. In the introductory figures the authors detail the relationship between MET (mean) and JSD (variance) for single sites. A similarly careful approach to understanding the relationship between mean and variance for regions was not taken. Because mean and variance are often correlated, I'm concerned that their interpretations of changes of JSD for particular genomic regions is entirely dependent on the mean DNA methylation levels of these regions.

It is true that MET and JSD are correlated and we have discussed this correlation at the genome-wide level in the submitted manuscript. In general, we expect that JSD increases if methylation is increased for low-methylated regions (i.e. positive correlation), but we expect that JSD also increases if MET is decreased in highly methylated regions (i.e. negative correlation). We have in fact analyzed the relationship between MET and JSD for all types of regions that were mentioned in the manuscript. That is, we have phase plane plots for these regions including calculations of Spearman’s correlation coefficient (and p-values) in the data repository accompanying the paper. One example was given in the supplemental Figure S9 for CMT2- and RdDM-targeted TEs, which we now decided to move to the main text. But we also want to point out that the data clearly shows that for a given MET (except for MET=0 and MET=1 where JSD=0), there is considerable variation possible in JSD (see also our revisions in the text addressing comment 23). The extreme case is obviously MET=0.5, where JSD can vary between 0 and 1. A related aspect that we now discuss more thoroughly is the distinction between regions with high JSD and regions that have an increase in the average site-specific JSD. Only the latter has been analyzed here. See also comment 3 and the paragraph in the discussion starting with “*Different approaches already exist to detect differentially methylated cytosines (DMCs)…*”

18. The authors did not explore the use of their statistic between tissue types. How does variance between tissue compare to variance within tissue?

We agree that this is an obvious question. As we have stated in the discussion: “A serious limitation of the implementation of JSD in its current form, however, is that it only addresses within-population diversity. The extension of JSD to more levels of population subdivision, similar to hierarchical F-statistics [1, 53] is outstanding but it would allow us to apportion diversity to diﬀerent levels. This would increase the practical value of the JSD-based approach to detecting diversity at genome scale.” That is, quantifying JSD between tissues is unfortunately not possible with the current state of development of JSD for methylation divergence.

## Other comments

19. Other Comments: Page 1, Column 1, Line 47. "The translation of these population level approaches to genome-wide chromatin marks is still missing, but the data available…" I believe there are missing reference here. Others have attempted to apply a population genetic framework to interpret sites with high mutability, like DNA methylation status (Charlesworth, Genetics, 2014; Wang, MBE, 2015; Van der Graaf, 2015, PNAS)

We thank the reviewer for pointing out this omission as it allows us to clarify what we mean here. We do not claim that the study of variation of epigenetic marks requires adopting a population genetics framework. We were under the impression that diversity is a generic concept that can and is also studied at the phenotypic level. We have changed the wording and added references to avoid misunderstandings.

20. Page 2, Column 1, Line 26 "Due to its correlation with fitness relevant traits … soft inheritance". Please add citation.

We have added relevant citations.

21. Please explain how the empirical cutoffs for MSCs were defined in Fig 1B. Why was this determined by the vertex at MET 20/80% with H[P]?

This is now explained in the main text in the first paragraph of the subsection “*Divergence is higher in the CG than the non-CG context”.*

22. It could be helpful to highlight MSC sites for each context in Fig 2A, either with a line, or colored points.

We have added lines and annotations to help with the orientation.

23. Page 2, Column 2, Line 58. "The C-sites with low to moderate JSD are mainly LMCs, as expected for the largely unmethylated genome of Arabidopsis". The figures are not referenced for many of the sentences interpreting the data and made it difficult to follow. This is 1 example. I think this should be referencing Fig 2B, but I don't see evidence for differences in JSDs.

We thank Reviewer #2 for pointing out these confusing parts of the manuscript. We have rewritten this paragraph to clarify the connection between text and figures. Also, because there are no editorial restrictions, we have decided to move the table comprising the C type proportions, which was in the supplement, to the main text. We think that this is one of the cases where a table alongside the text works better than a figure. We have also divided this paragraph into two, the new paragraph now dealing only with the correlation between MET and JSD and added the corresponding figure in the supplement to the main text as well.

24. Page 5, Column 1, Line 8 - "In fact, the joint distribution … HMC's". No figure cited here again.

Figure reference has been added.

25. Page 5, Column 1, Line 19- "genotype - and cell-type specific regulation play the main role in shaping the methylation landscape". No citation listed and the variation between genotypes was not analyzed in this paper.

We have modified the wording to emphasize that we speculate here about the reason for the observed stability, but we have also added a citation to a relevant review.

26. Page 5, Column 1, Line 23 "Non-CG sites segregate within rather than among individuals" - No figures were cited in this section. Please reference the appropriate data.

We have removed the entire subsection (see comments 7 and 27).

27. Page 5, Column 1, Line 28. "First of all, a low JSD for a given site means that there is weak segregation across the population. That is these sites show intermediate methylation levels in the majority of individuals". The term "segregation" is not appropriate for tissue variation in methylation levels. Additionally, this entire section is speculative as the inference of methylation on only the maternal or paternal copies is not supported. Strand specificity or unbiased sampling at these sites compared to the genome-wide background was not determined. This section should be removed, or data added to support the proposed interpretation.

We have removed the entire subsection because it turns out to be rather confusing and speculative (see also comments 7 and 26). We have added a brief discussion of the intermediate MET levels to the previous subsection.

28. Fig 2E - Please explain chromosome track colors and or axes. Why is CG plotted on the positive y-axis and non-CG plotted on the negative y-axis. Why are CHG/CHH stacked? Does this include MSC?

See also comment 8. This is just a stacked bar chart, there is no positive/negative axis involved but the stacking is centered. All three contexts are stacked for compact illustration. The color code is the same throughout the paper for the three contexts and their respective C types which we have now emphasized in the figure legend. The MSCs are better visible in the zoom-in view (see comment 8).

29. Page 6, Column 1, Line 42 - "we ask how much diversity in DNA methylation certain regions can tolerate without their chromatin state being affected". Cause/effect relationships between chromatin status and DNA methylation cannot be tested with this data set.

This sentence has been rephrased to avoid misunderstanding: “*In this section, we want to investigate whether regions that have a different chromatin state also differ with respect to methylation divergence.”*

30. Fig 3A - It is stated that MET and JSD are correlated, but subtle differences were discussed. Were there any statistical tests done to determine if difference existed?

This is a misunderstanding and we have tried to correct the wording in the paragraph. For some states we see increases in both MET and JSD but that does not necessarily mean a positive correlation. We also have done chromatin state-specific phase plane plots that includes a test for correlation based on Spearman’s correlation coefficient similar to Fig 2a (see also our answer to comment 17). This data is, partially, in the accompanying Zenodo repository. For example, for the heterochromatic state 8 we have in the CG context differing correlations for different sources:

*plot-phaseplane_chromatin-state8_CpG_Col-0_wt_aerial-part.svg:*

*spearmanr = -0.36; p = 0*

*plot-phaseplane_chromatin-state8_CpG_Col-0_wt_embryo.svg:*

*spearmanr = -0.48; p = 0*

*plot-phaseplane_chromatin-state8_CpG_Col-0_wt_endosperm.svg:*

*spearmanr = 0.064; p = 3.3e-101*

*plot-phaseplane_chromatin-state8_CpG_Col-0_wt_immature-flower-buds.svg:*

*spearmanr = -0.71; p = 0*

*plot-phaseplane_chromatin-state8_CpG_Col-0_wt_inflorescence.svg:*

*spearmanr = -0.08; p = 0*

*plot-phaseplane_chromatin-state8_CpG_Col-0_wt_root.svg:*

*spearmanr = 0.064; p = 0*

*plot-phaseplane_chromatin-state8_CpG_Col-0_wt_rosette.svg:*

*spearmanr = -0.13; p = 0*

*plot-phaseplane_chromatin-state8_CpG_Col-0_wt_shoot.svg:*

*spearmanr = 0.034; p = 2.7e-94*

*plot-phaseplane_chromatin-state8_CpG_Col-0_wt_sperm-cell.svg:*

*spearmanr = -0.35; p = 0*

*plot-phaseplane_chromatin-state8_CpG_Col-0_wt_vegetative-nucleus.svg:*

*spearmanr = -0.11; p = 0*

*plot-phaseplane_chromatin-state8_CpG_Col-0_wt_whole-organism.svg:*

*spearmanr = -0.15; p = 0*

31. Page 8, Column 1, Line 13 -" With respect to the levels of JSD … we observe the order S7 > S3 > S6)". Are these values the average across the regions? Was a statistical test done to determine difference?

Yes, these are comparisons of the averages. No statistical significance test has been done. We have rewritten this part slightly to clarify the discussion.

32. Figure 3C. H3K9me2 is the most correlated with JSD, but not discussed in the text as much as the other marks.

We have added more explanations regarding this histone mark in the relevant section on chromatin states.

33. Page 9, Column 1, Row 13 - "TEs can provoke higher methylation diversity". This title again implies causality, but causality cannot be determined.

We have changed the title to “*TEs are associated with increased methylation diversity”.*

34. Please define TEG more carefully. Does this refer to GAG/POL etc?

We have used the definition of TAIR for transposable element genes (TEGs) as this has also been used in the Araport 11 release. This is now mentioned and better described in the text.

35. Page 9, Column 1, Row 30 - "The JSD profile … although the asymmetric bell shape is less pronounced". The bell pattern is not apparent in Fig 4.

We have changed the sentence slightly. The asymmetry for methylation is well known and visible in all published metagene profiles but not so visible here because we decided to draw the profiles with heatmaps for a compact representation of all the information.

36. Page 9, Column1, Row 59 - "In summary, TEs are clearly associated with higher methylation diversity in genes … in and around genes makes sense". I'm concerned that MET and JSD in regions are highly correlated.

See our answer to previous comments regarding this issue and also the Figure with the phase plane plot of RdDM- and CMT2-targeted TEs.

37. Fig 6B - I could not follow the interpretation in the text for this figure and was not able to identify the relationship between MSG and TE type.

We have added more explanations for this type of plot in this section to clarify its interpretation.

38. Section of gene expression differences: The authors analyze the 50 Top/bottom expressed genes for each tissue and then attempt to understand variation in gbM at these genes. In A. thaliana, gbM is typically enriched in moderately to moderately/highly expressed genes and then drops off for the most highly expressed genes in the sample. I'm concerned that their conclusions that there is a "dubious relationship" between gBM and expression is true for these 50 genes, but that these genes aren't representative of typically gbM genes.

We performed a correlation analysis of gene expression with MET/JSD and found that there is indeed a negative correlation of gene expression and MET/JSD, that is, when picking a random gene with high average JSD, it is likely to be repressed or to have very low expression. We have added this analysis to the supplement, changed our conclusion slightly, and added another citation. While the correlation may be real, we still question the general significance of this observation because there are so many counterexamples.

39. Page 15, Column 2, Row 7 - "meta-methylome" term is referenced. I'm not convinced that a new term for studying mean and variance of DNA methylation is warranted here.

After re-considering this term, we agree with Reviewer #2. We have removed it from the text as it does not enlighten any of the findings.

40. Methods - Please explain the weights for the JSD statistic more clearly in the methods.

We have added more explanations in the Methods section and also discuss the importance of weights for estimation in the Discussion.

**Second round of review**

**Reviewer 1**

The authors have answered all my questions. One small issue is that Table 2 is cited before Table 1.

**Reviewer 2**

After re-reviewing the submitted manuscript by Kartal et. al., I find that the authors have made serious efforts to address my original concerns. The revised manuscript articulates the main findings clearly and attempted to make their analysis accessible to a more general audience. Additionally, caveats with their approach were more clearly outlined in the text. I have no major concerns but will highlight one lingering comment.

One of my original concerns was that the authors sought to interpret their summary statistics using population genetic theory and that variance in the level of DNA methylation between samples (not even genetically distinct) cannot be interpreted using population genetics. The authors removed or rephrased most of these issues through the text, but there are still misleading statements in the abstract and introduction.  It is reasonable to discuss future steps that may be taken to assess genetically diverse samples, but this should be limited to the discussion in my opinion. The text of concern are the first 2 sentences of the abstract and the second half of the first paragraph starting with "For example, genome scans …".

**Authors Response**

**Point-by-point responses to the reviewers’ comments:**

Reviewer #1: The authors have answered all my questions. One small issue is that Table 2 is cited before Table 1.

*We thank the reviewer for spotting this error, we now refer to Table 2 explicitly only in the Methods section which fixes this problem.*

Reviewer #2: After re-reviewing the submitted manuscript by Kartal et. al., I find that the authors have made serious efforts to address my original concerns. The revised manuscript articulates the main findings clearly and attempted to make their analysis accessible to a more general audience. Additionally, caveats with their approach were more clearly outlined in the text. I have no major concerns but will highlight one lingering comment.

*We thank the reviewer for this appreciation and the constructive criticism which helped to improve our manuscript.*

One of my original concerns was that the authors sought to interpret their summary statistics using population genetic theory and that variance in the level of DNA methylation between samples (not even genetically distinct) cannot be interpreted using population genetics. The authors removed or rephrased most of these issues through the text, but there are still misleading statements in the abstract and introduction. It is reasonable to discuss future steps that may be taken to assess genetically diverse samples, but this should be limited to the discussion in my opinion. The text of concern are the first 2 sentences of the abstract and the second half of the first paragraph starting with “For example, genome scans …”.

*We have rephrased the relevant parts, but we still think it is useful and correct to point out the analogy between genome and epigenome scans as methods.*
